# The structure and assembly of reaction centre-light-harvesting 1 complexes in photosynthetic bacteria

**DOI:** 10.1042/BSR20220089

**Published:** 2023-05-25

**Authors:** David J.K. Swainsbury, Pu Qian, Andrew Hitchcock, C. Neil Hunter

**Affiliations:** 1 School of Biosciences, University of Sheffield, Sheffield, UK; 2 School of Biological Sciences, University of East Anglia, Norwich, UK; 3 Materials and Structural Analysis, Thermo Fisher Scientific, Eindhoven, Netherlands

**Keywords:** bacteriochlorophyll, carotenoids, cryogenic electron microscopy, light-harvesting complex, photosynthesis, purple bacteria

## Abstract

Chlorophototrophic organisms have a charge-separating reaction centre (RC) complex that receives energy from a dedicated light-harvesting (LH) antenna. In the purple phototrophic bacteria, these two functions are embodied by the ‘core’ photosynthetic component, the RC-LH1 complex. RC-LH1 complexes sit within a membrane bilayer, with the central RC wholly or partly surrounded by a curved array of LH1 subunits that bind a series of bacteriochlorophyll (BChl) and carotenoid pigments. Decades of research have shown that the absorption of light initiates a cascade of energy, electron, and proton transfers that culminate in the formation of a quinol, which is subsequently oxidized by the cytochrome *bc*_1_ complex. However, a full understanding of all these processes, from femtosecond absorption of light to millisecond quinone diffusion, requires a level of molecular detail that was lacking until the remarkable recent upsurge in the availability of RC-LH1 structures. Here, we survey 13 recently determined RC-LH1 assemblies, and we compare the precise molecular arrangements of pigments and proteins that allow efficient light absorption and the transfer of energy, electrons and protons. We highlight shared structural features, as well as differences that span the bound pigments and cofactors, the structures of individual subunits, the overall architecture of the complexes, and the roles of additional subunits newly identified in just one or a few species. We discuss RC-LH1 structures in the context of prior biochemical and spectroscopic investigations, which together enhance our understanding of the molecular mechanisms of photosynthesis in the purple phototrophic bacteria. A particular emphasis is placed on how the remarkable and unexpected structural diversity in RC-LH1 complexes demonstrates different evolutionary solutions for maximising pigment density for optimised light harvesting, whilst balancing the requirement for efficient quinone diffusion between RC and cytochrome *bc*_1_ complexes through the encircling LH1 complex.

## Introduction

Photosynthesis converts the energy of sunlight to a charge separation, then to a proton-motive force that generates ATP. Ultimately, solar energy drives the formation of biomass from the reductive fixation of carbon dioxide. In phototrophic bacteria, algae and plants the first steps of photosynthesis rely on pigment-protein complexes, which absorb light and transfer the energy to a reaction centre (RC) complex [1,2]. Here, the absorbed energy arrives at a specialised set of pigments that undergo a charge separation; as a result, an electron passes along a series of redox-active carriers until it reaches a quinone or an iron-sulphur centre that acts as an electron acceptor [3]. This review focuses on the RC-light-harvesting 1 (RC-LH1) pigment-protein complexes of the purple phototrophic bacteria, which represent a relatively simple, modular solution to the problem of collecting and storing solar energy. Nature’s detailed blueprint for surrounding a charge-separating RC with a curved LH1 array of light-absorbing bacteriochlorophyll (BChl) and carotenoid chromophores, like a satellite dish enclosing a receiver, has been revealed only recently following years of research by many laboratories. As a result, several RC-LH1 structures have emerged with a resolution sufficient to show the positions of all pigments and protein side chains. Detailed descriptions of pigment–protein and pigment–pigment interactions for all of these complexes are beyond the scope of this review and this information can be found in the original reports of the respective structures. Instead, this review will survey the current range of RC-LH1 structures, drawing attention to shared structural themes and motifs, and highlighting their numerous and fascinating differences.

The recent upsurge in RC-LH1 structures arises from advances in cryogenic electron microscopy (cryo-EM), first applied to the complex from *Blastochloris* (*Blc*.) *viridis* [4]. Previously, X-ray crystallography had provided the only route to high structural resolution [5], following earlier lower-resolution crystallographic studies [6,7]. Previous EM analyses of RC-LH1 complexes had focused on two-dimensional crystals [8–11], or on single particles [12], revealing projection structures with resolutions in the 8–12 Å range. Indeed, atomic force microscopy (AFM) was at least as informative in terms of showing the basic architecture of RC-LH1 complexes [13], often with the bonus of showing their arrangement in the native photosynthetic membrane [14,15].

**Figure 1:**
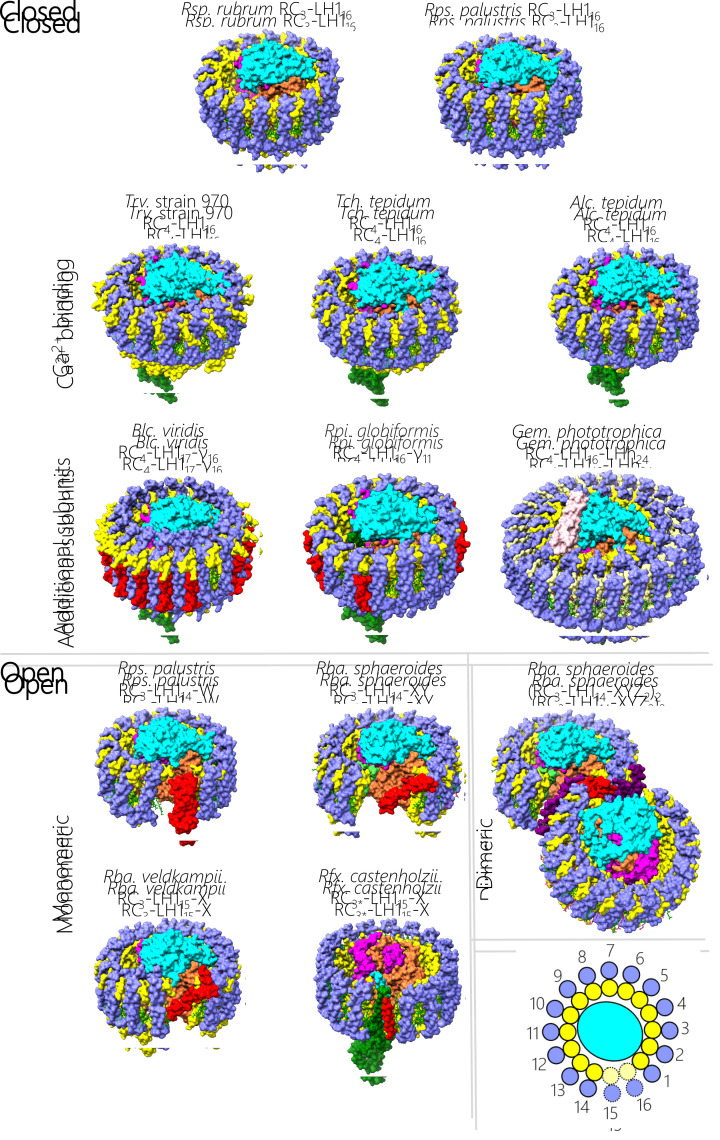
Overall architecture of RC-LH1 complexes available in the protein data bank Tilted views of RC-LH1 complexes with the protein in surface representation (RC-L in orange, RC-M in magenta, RC-H in cyan, RC-C in green, LH1 α in yellow, LH1 β in blue, and additional peptides in red, light green, purple or pink). Pigments and cofactors are shown in stick representation with BChls in green, carotenoids in purple, hemes in red, and quinones in yellow. The complexes from various bacterial species are grouped according to their structural features, using a standardised name that describes their composition. The bottom right panel shows the scheme for numbering LH1 subunits, with dotted lines used to denote missing subunits that create a gap in the ring. Note that the *Rfx. castenholzii* RC lacks an H-subunit, so it is a three-subunit RC with properties more like that of a four-subunit RC. Therefore, we have denoted it RC_3_*.

**Figure 2:**
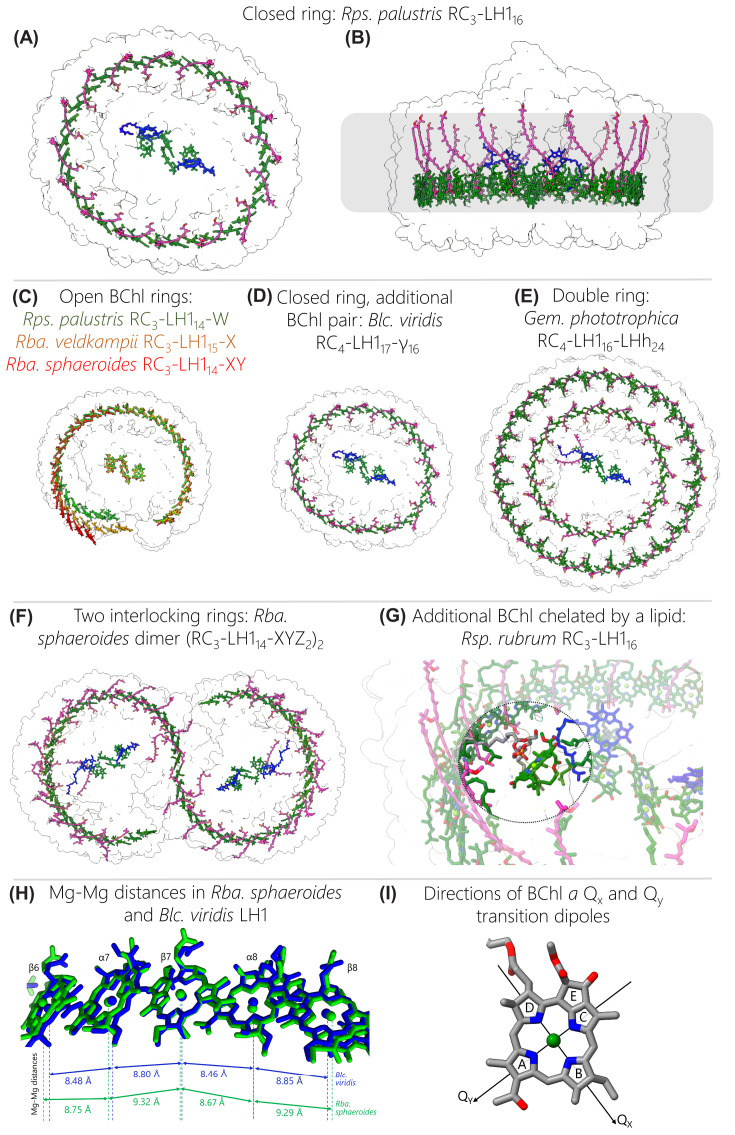
Arrangements of bacteriochlorophylls and carotenoids in RC-LH1 complexes (**A**) View of the cytoplasmic face of a typical closed RC-LH1 pigment arrangement, in this case the *Rps. palustris* RC-LH1_16_ complex. The protein is outlined in white to highlight the pigments; BChls *a* are in green, bacteriopheophytins (BPhe) are in blue, and carotenoids are in magenta. (**B**) View in the plane of the membrane, which is represented by a grey rectangle. (**C**) Superimposed arrangement of BChls in open RC-LH1 complexes from *Rps. palustris* in green, *Rba. veldkampii* in orange, and *Rba. sphaeroides* in red. The white outline is that of the *Rps. palustris* RC_3_-LH1_14_-W complex. (**D**) Pigments in the *Blc*. *viridis* RC_4_-LH1_17_-γ_16_ complex, with the same colour coding as in (**A**) but in this case green denotes BChl *b* and blue BPhe *b*. (**E**) Pigments within the *Gem. phototrophica* double-ring complex. (**F**) Pigments within the dimeric *Rba. sphaeroides* (RC_3_-LH1_14_-XYZ_2_)_2_ complex. (**G**) Zoomed view of the additional BChl (dark green) chelated by a lipid (grey) on the inner face of the LH1 complex of *Rsp. rubrum*. (**H**) Enlarged view of aligned BChls from *Rba. sphaeroides* (green) and *Blc. viridis* (blue). Each BChl is annotated with the subunit that chelates its central Mg atom, and Mg-Mg distances are shown with dashed lines and labelled arrows. BChls are aligned to the nitrogen atoms of BChl β7. (**I**) A BChl *a* molecule annotated with ring letters and the directions of the Q_x_ and Q_y_ transition dipoles The majority of the phytol tail on ring D has been removed.

**Figure 3:**
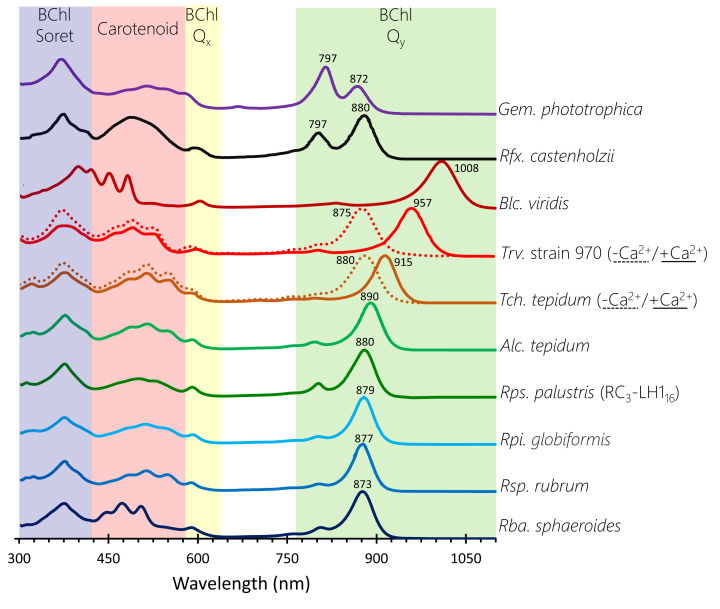
Absorption spectra of RC-LH1 complexes The coloured spectral regions denote the approximate spectral coverage for BChl Soret, carotenoid, BChl Q_x_ and BChl Q_y_ absorption. The spectra are from the authors of this study, with the exceptions of *Blc. viridis* and *Rsp. rubrum* (Dr Daniel Canniffe, University of Liverpool), and are otherwise adapted from relevant publications for *Trv*. strain 970 [28], *Tch. tepidum* [29], *Alc. tepidum* [30], *Rpi. globiformis* [31] and *Rfx. castenholzii* [69]. These spectra are of complexes solubilised in detergents, which are blue-shifted relative to their spectra when in native biological membranes.

**Figure 4:**
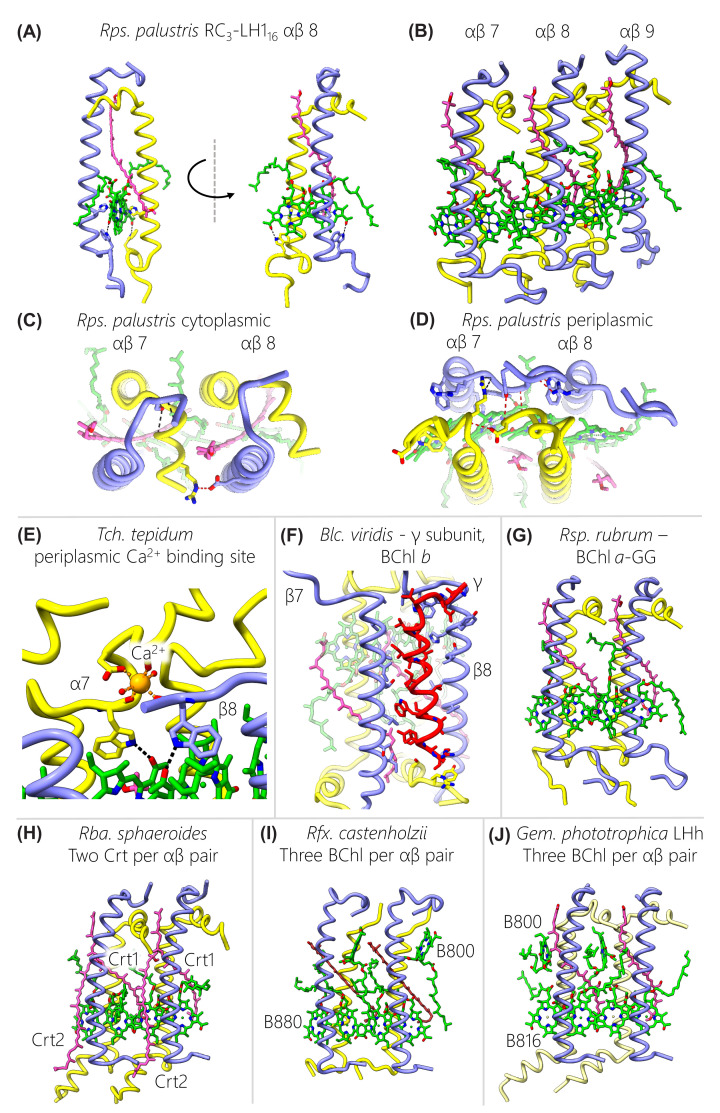
Structures of the αβBChlCrt units that associate to form LH1 complexes (**A**) One αβBChl_2_Crt unit from *Rps. palustris*, which represents the most common structure observed in LH1 complexes. (**B**) An array of three αβBChl_2_Crt units showing how they associate to form an LH1 ring. (**C,D**) Expanded views of the cytoplasmic and periplasmic regions of two αβBChl_2_Crt units showing the inter-subunit (red) and intra-subunit (black) hydrogen bonds that help stabilise the complex. (**E**) Enlarged view of the Ca^2+^ binding site that adds additional inter-subunit interactions between αβ pairs in *Tch. tepidum* LH1. (**F**) Enlarged view of the γ-subunit of *Blc. viridis* showing its interactions with the TMHs of two adjacent β-subunits. (**G**) Two αβBChl_2_Crt units from *Rsp. rubrum* binding BChl *a* with a GG tail instead of the phytol tail (as in the other examples). (**H**) Two αβBChl_2_Crt_2_ units from *Rba. sphaeroides* showing the incorporation of a second carotenoid that binds between β-subunits. (**I**) Two αβBChl_3_Crt units from *Rfx. castenholzii* showing the addition of a monomeric BChl towards the cytoplasmic face of each subunit. (**J**) Two αβBChl_3_Crt units from the outer LHh ring of *Gem. phototrophica*, which bind a BChl dimer absorbing at 816 nm and a monomer absorbing at 800 nm.

**Figure 5:**
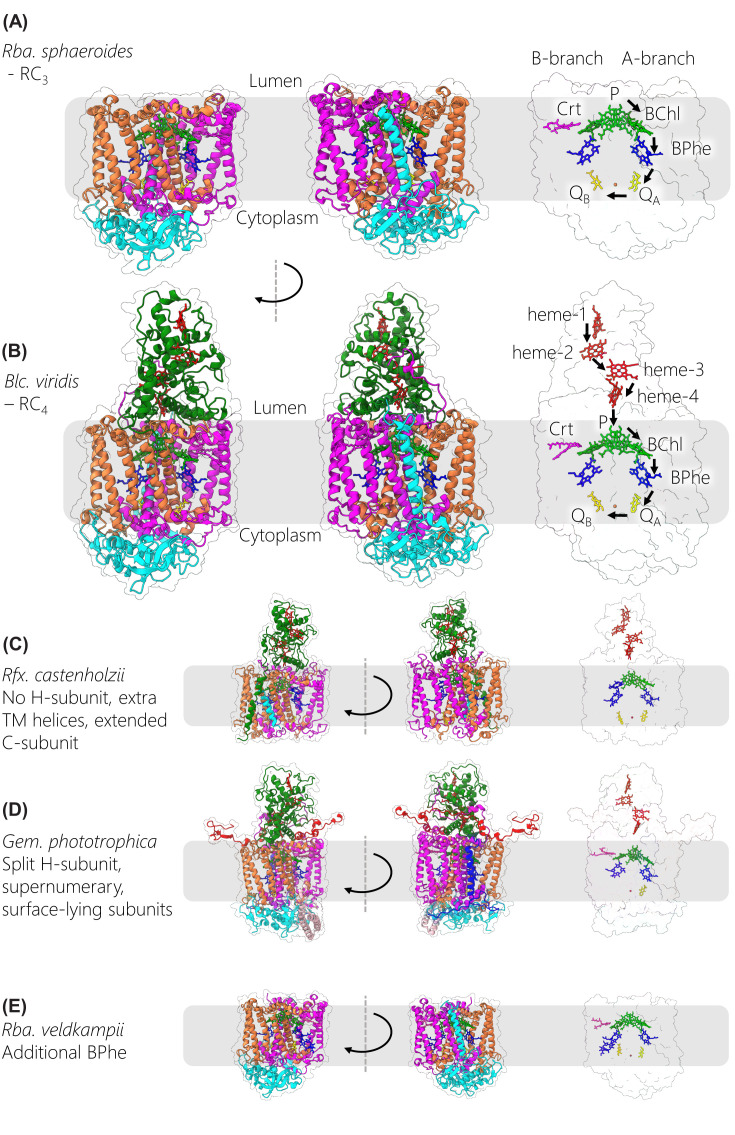
Structure and cofactor organisation of bacterial reaction centres (**A**) Crystal structure of the three-subunit RC from *Rba. sphaeroides* (PDB ID:2J8C) shown in two orientations, and a view with proteins in semi-transparent representation to reveal the bound cofactors with black arrows indicating electron transfer steps. (**B**) Crystal structure of the four-subunit RC from *Blc. viridis* (PDB ID: 1PRC), which also binds a tetraheme cytochrome C-subunit, in the same orientations as in (A). (**C**) The three-subunit RC of *Rfx*. *castenholzii* which has no H-subunit, an additional TMH, and a TMH anchor on its C-subunit. (**D**) The *Gem*. *phototrophica* RC, which resembles a four-subunit RC with a split H-subunit (cyan, blue) and several supernumerary subunits. (**E**) The three-subunit RC of *Rba. veldkampii*, which binds an additional BPhe. (C–E) are from the respective RC-LH1 structures. Colours: RC-L (orange), RC-M (magenta), RC-H (cyan), RC-C (green). In (C) the additional TMH-X is in cyan; in (D) the supernumerary subunits are in red (RC-S) and dark pink (RC-U).

**Figure 6:**
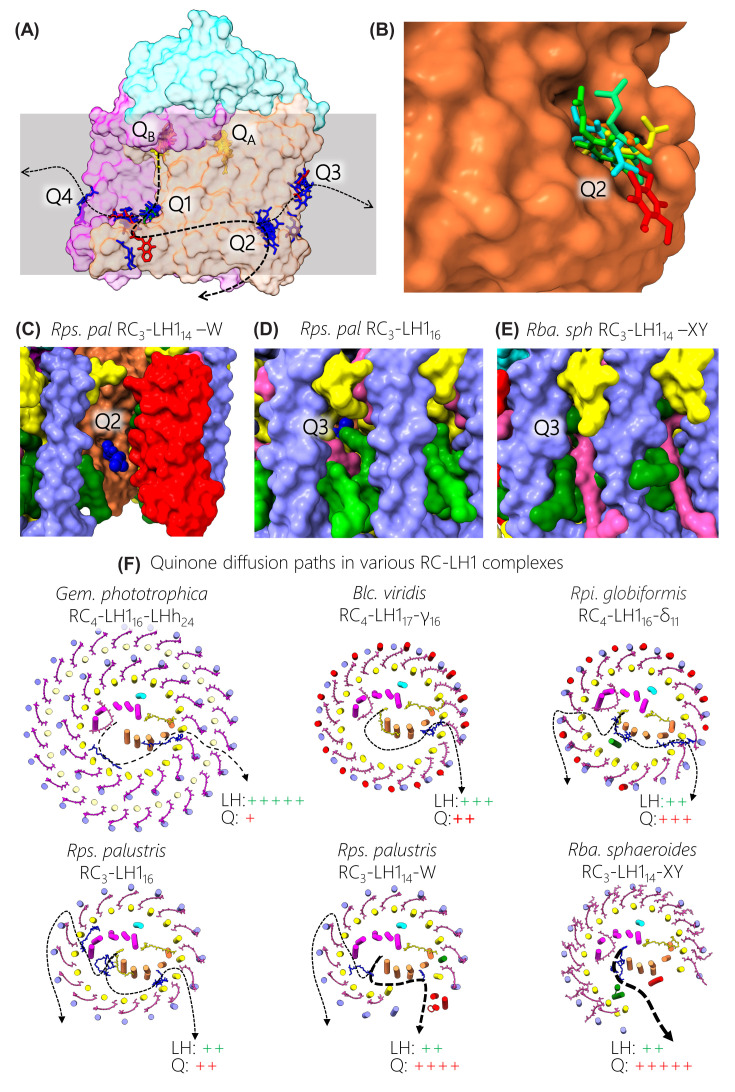
Quinone diffusion through RC-LH1 complexes (**A**) Semi-transparent surface view of the RC from *Rps. palustris* with overlaid accessory quinones from all structures. Clusters of resolved quinones are labelled Q1-4. The Q_A_ and Q_B_ quinones are in yellow, accessory ubiquinones are in blue, menaquinones in red and rhodoquinone is in green. For clarity isoprene trails have been truncated to one unit. (**B**) Enlarged view of site Q2 with the quinones from different structures in unique colours. (**C**) The gap in the *Rps. palustris* RC-LH1_14_-W complex with its Q2 quinone shown in spacefill representation. (**D**) View of the narrow pores through which quinone is expected to diffuse between αβ pairs at the Q3 position of the *Rps. palustris* RC-LH1_16_ complex. The Q3 quinone in spacefill representation is from the *Tch. tepidum* complex. (**E**) A view adjacent to Q3 in the *Rba. sphaeroides* RC-LH1 complex showing how the second carotenoid obscures the pore through which quinones can diffuse. (**F**) Views of various complexes perpendicular to the plane of the membrane, simplified by omitting BChls, by using coloured circles for transmembrane helices, and with stick representations for carotenoids and quinones. Dashed arrows show potential routes for quinone diffusion with each line thickness providing a rough indication of the likelihood for each pathway. The LH and Q +/- designations are for comparative purposes, as rough indicators of the capacity of each complex for light harvesting and quinone traffic.

**Figure 7:**
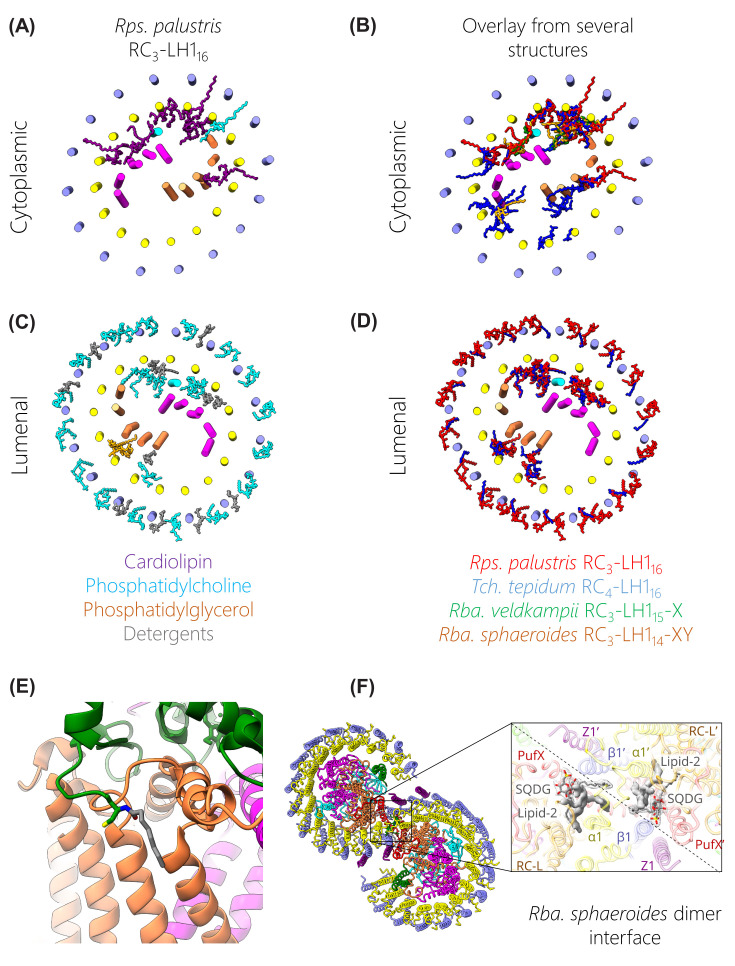
Resolved lipids in RC-LH1 complexes (**A**) Lipids on the cytoplasmic face in the *Rps. palustris* RC-LH1_16_ complex (coloured as in key in panel C). (**B**) Overlaid cytoplasmic lipids from selected complexes (see key in D). (**C**) Lipids from the lumenal face of the *Rps. palustris* RC-LH1_16_ complex. (**D**) Overlaid lipids in four RC-LH1 complexes, viewed from the lumenal face. (**E**) Modification of the N-terminus of Cys23 (following removal of the signal peptide) in the *Tch. tepidum* complex, providing a lipid anchor (grey) for the RC-C subunit (green). (**F**) SQDG lipids at the dimer interface in the *Rba. sphaeroides* (RC_3_-LH1_14_-XYZ_2_)_2_ complex. The interface is outlined with a black square and the expanded view shows the interface from the periplasmic side, using a diagonal dashed line to indicate the approximate position of the interface between monomers. For clarity, BChl and carotenoid pigments have been omitted. SQDG lipids are shown within a mesh representing the density; the solid grey densities for another, unassigned, lipid (Lipid-2) are also shown. Polypeptides belonging to each monomer half of the complex are labelled as RC-L, RC-L’, for example.

**Figure 8:**
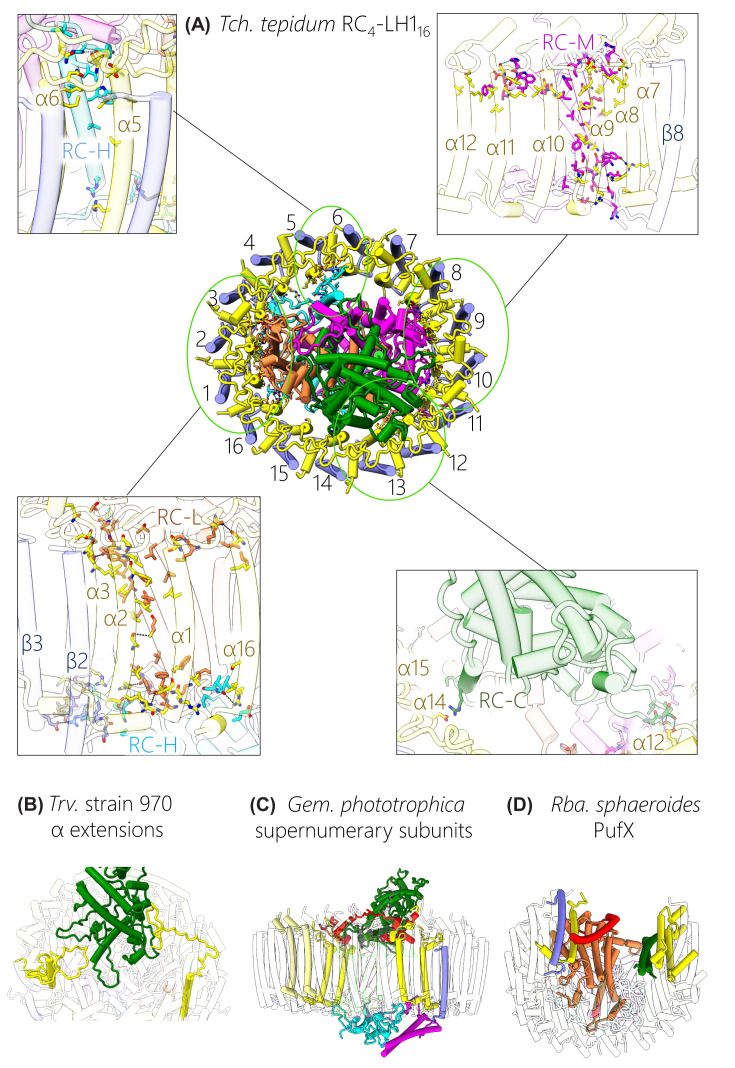
Protein–protein interactions between the RC and LH1 (**A**) View of the *Tch. tepidum* RC-LH1_16_ complex from the periplasmic face with residues mediating RC-to-LH1 interactions shown as sticks. Regions of interest in the green ellipses are expanded and reoriented for clarity in the four boxes. Interacting sidechains are in solid colour and protein subunits are in semi-transparent colour. (**B**) Expanded view of periplasmic C-terminal extensions to the LH1α polypeptides that provide additional interactions between LH1 and the C-subunit of the RC in *Trv*. strain 970. (**C**) Supernumerary subunits in the *Gem. phototrophica* complex (RC-S in red, RC-U in magenta) that interact with the RC C- and H-subunits, and with the LH1 ring (specific LH1 polypeptides in yellow) on both faces of the complexes. (**D**) PufX in the *Rba. sphaeroides* complex (red) interacting with the RC L-subunit, and the first αβ pair of LH1. Protein-Y is in green. This tilted view of the cytoplasmic side of the complex shows the opening in the LH1 ring. In (B–D) interacting subunits are in solid colour and other protein subunits are in white.

The RC-LH1 complexes of purple phototrophs all consist of a belt of light-absorbing BChl and carotenoid pigments, held in place by a repeating series of transmembrane polypeptides that curve tightly round a central RC [16,17]. The absorption behaviour of these pigments is tuned by exciton coupling and hydrogen bonding, ensuring that light is collected over a wide spectral range that spans the near-ultraviolet, visible and near-infrared regions of the spectrum. This energy passes to the RC, initiating a series of electron transfers that culminate in reduction of a quinone acceptor. After two rounds of charge separation the quinone is doubly reduced, and after binding two protons from the cytoplasm it becomes a quinol, which leaves the RC and diffuses to a cytochrome (cyt) *bc*_1_ complex (or cyt *b*_6_*f* complex in cyanobacteria, algae and plants). Here, the quinol releases its cargo of electrons and protons and the resulting quinone makes the return trip to the RC. Meanwhile, having been reduced by cyt *bc*_1_, a cyt *c*_2_ on the lumenal side of the RC donates an electron and fills the ‘hole’ generated by photo-oxidation of the RC, resetting the system for another round of photochemistry. This review highlights recent advances in structural analysis of RC-LH1 complexes, which reveal the detailed basis for light absorption, excitation energy transfer, charge separation, electron transfer, and finally production and export of quinols. These structures illustrate several strategies for optimising the absorption of light by packing more pigments round the RC while allowing quinones and quinols access to the RC Q_B_ site, which requires gaps in the LH1 ring. Figure 1 compares the overall architecture of the 13 RC-LH1 complexes deposited in the PDB at the time of writing, then Figures 2-8 deal with the absorption and transfer of light energy, a comparison of the various RCs, quinone traffic across the LH complexes, lipids, RC-LH1 interactions, and finally assembly of the RC-LH1 complex.

## The photosynthetic membranes that contain RC-LH1 complexes

In most natural environments, phototrophs need to harvest as much solar energy as possible and, given that (B)Chl-binding complexes are generally embedded in a membrane bilayer, they adopt several strategies to optimise the membrane surface area. Tomographic studies of purple phototrophs, cyanobacteria and chloroplasts have revealed the proliferation and complexity of these internal membranes [18–21]. In the case of the model cyanobacterium *Synechocystis* sp. PCC 6803, for example, a single cell of ∼2 μM diameter can accommodate up to 19 μM^2^ of thylakoid membrane sheets, packed with photosystem I and II complexes, as well as with other complexes involved in electron transfers [22,23]. The same principles apply to purple phototrophic bacteria such as *Rhodobacter* (*Rba*.) *sphaeroides* [19], which can contain over 1,000 membrane vesicles of ∼50 nm diameter representing 16 μM^2^ of membrane area for harvesting and utilising solar energy [24]. In some bacterial phototrophs, for example *Rhodospirillum* (*Rsp*.) *rubrum*, these intracellular vesicles largely consist of RC-LH1 complexes whereas many other bacteria have additional light-harvesting components. These peripheral LH2 antenna complexes provide an economical way of adding more light-absorbing capacity because they bind approximately 2.5-fold more BChls than RC-LH1 complexes per nm^2^ of membrane area. Furthermore, high levels of light suppress assembly of the peripheral antenna network, as well as constraining the assembly of membrane vesicles, providing two levels of control in response to increased light intensity [24]. Thus, cells only incur the metabolic cost of synthesising these complexes when they are needed.

## A survey of the architecture of RC-LH1 complexes

The RC-LH1 complex is the essential component of bacterial photosynthesis, and its basic architecture consists of a central, charge-separating RC wholly or partly encircled by a light-harvesting system. These RC-LH1 assemblies are frequently called ‘core’ complexes because they are the primary photochemical complex in purple phototrophic bacteria. Figure 1 displays thirteen RC-LH1 structures from a variety of phototrophic bacteria, using a consistent colour coding for RC and LH1 subunits to emphasise the similarities between complexes. Nonetheless, the differences between complexes are immediately apparent, with closed rings found in *Rsp. rubrum* [25,26], *Rhodopseudomonas* (*Rps*.) *palustris* [27], *Thiorhodovibrio* (*Trv*.) strain 970 [28], *Thermochromatium* (*Tch*.) *tepidum* [29], *Allochromatium* (*Alc*.) *tepidum* [30], *Blastochloris* (*Blc*.) *viridis* [4], *Rhodopila* (*Rpi*.) *globiformis* [31], *Gemmatimonas* (*Gem*.) *phototrophica* [32], and with incomplete, open rings in *Rps. palustris* [27], *Rba. sphaeroides* [33], *Rba. veldkampii* [34] and *Roseiflexus* (*Rfx*.) *castenholzii* [35]. Within these categories, there are major modifications in the case of the double LH ring in the *Gem. phototrophica* complex [32], and with the dimeric core structure of *Rba. sphaeroides* [36–38]. To establish a nomenclature sufficient to encompass these diverse RC-LH1 structures, we distinguish between central RC_3_ and RC_4_ complexes consisting of three (H (cyan), L (orange), M (magenta)) or four (H, L, M, C (green)) subunits, respectively. The RCs are enclosed by a curved array of LH_*n*_ subunits, each consisting of an α (yellow)-β (blue) pair of transmembrane polypeptides. The LH1 subscript *n* denotes the number of subunits, with at least 16 α and 16 β in closed LH1 antennas, or 14 or 15 αβ subunits in complexes with open rings.

Some LH1 complexes are embellished with extra components, coloured red, light green, purple or pink in Figure 1. For example, in *Blc. viridis* the αβ_17_ LH1 complex is augmented by an outer ring of 16 γ-polypeptides that make extensive contacts, predominantly with the outer ring of β-polypeptides; these interactions were suggested to stabilize the LH1 structure and contribute to the large red-shift in absorption for this complex [4]. Genetic removal of γ-polypeptides, followed by their reinstatement, recently verified this role for the γ-subunit [39]. The absence of a 17th γ subunit creates a gap through which quinones can pass. In *Rpi. globiformis* there is a 16-subunit ring but only 11 γ-like polypeptides, creating more possibilities for diffusion of quinols and quinones [31]. The open ring complexes (Figure 1) all have at least one transmembrane component that prevents the LH1 complex from completely encircling the RC, maintaining a gap that allows the exchange of quinones and quinols to and from the RC Q_B_ site. Often, this component is a PufX or protein-W polypeptide, but in the case of *Rfx. castenholzii* the transmembrane domain of the cytochrome subunit (green) appears to interrupt the LH ring along with an additional transmembrane helix (TMH) called subunit X (red) [35]. The issue of quinone/quinol diffusion is crucial for the function of RC-LH1 complexes, yet the existence of closed structures shows that a gap in the LH1 ring is not strictly required. The issue of quinol export will be discussed in detail in a later section.

Special mention should be made of the remarkable double-ring complex in Figure 1, which was purified from an unusual bacterium, *Gem. phototrophica*, discovered in Swan Lake in the Gobi Desert [40]. This RC_4_-LH1_16_-LHh_24_ assembly is the largest of the RC-LH1 complexes, comprising 178 pigments bound to more than 80 protein subunits. No other such complexes have been found, possibly because of the distinct evolutionary pathway that led to its formation. *Gem. phototrophica* arose from non-phototrophic host that acquired the ability to photosynthesise following transfer of photosynthetic genes from an ancient phototrophic proteobacterium [40]. Subsequent evolution along an independent pathway resulted in *Gem. phototrophica* surrounding its RC with a novel, double ring light-harvesting system. The outer LHh ring augments the light-gathering capacity of the inner ring, which closely resembles the conventional LH1 complex seen in other closed ring systems. The light-harvesting mechanism will be discussed in more detail in the section on bacteriochlorophyll and carotenoid pigments, but here we note that many RC-LH1 complexes, such as those in *Rps. palustris* and *Rba. sphaeroides*, are augmented by other light-harvesting components. In such cases transmembrane LH2 rings consisting of 7-9 αβ polypeptides are under separate regulatory control with respect to RC-LH1 complexes, and 1-4 LH2 complexes can be packed against the outer face of each RC-LH1 complex, depending on the incident light intensity [24,41]. Some species produce spectral variants of the LH2 antenna, which provide additional adaptability to the changing spectral environments inhabited by these bacteria [42,43]. In *Gem. phototrophica* the outer LHh ring performs a function like that of LH2, but ‘hardwiring’ the outer and inner rings together represents a loss of adaptability in terms of responding to variable light levels.

The dimeric core complex from *Rba*. *sphaeroides* also stands out from the other examples in Figure 1. The formation of a dimer affects the membrane bilayer in which it sits, and there are also functional consequences in terms of energy trapping, which will be covered in the next section. The architecture of the dimer is interesting, because the cryo-EM structure of this complex shows that the two monomer halves subtend an angle of 152°. Earlier structures from EM of negatively stained complexes [12] and from X-ray crystallography [7] yielded angles of 146° and 158°, respectively. Thus, this is a membrane-bending complex that imposes local curvature, which can be propagated when several RC-LH1 dimers pack together in the photosynthetic membrane [44,45]. In the case of mutants with no LH2 complexes, the core dimers stack against one another in a way that produces elongated tubular membranes ∼72 nm in diameter and with micron-scale lengths [12,46–48]. The side-by-side packing of dimers along their long axes is offset, and the effect is to create a helical twist for each longitudinal array in which ∼73 RC-LH1 dimers are needed to complete one circle (360°) in the tubular photosynthetic membrane [12]. There are now three structures of the dimeric core complex from *Rba. sphaeroides* but Figure 1 shows 7PQD [36], which corresponds to 7VOR, one of two conformations resolved in a subsequent study [37].

In wild-type strains of bacteria such as *Rba. sphaeroides* the LH2 antenna is the dominant complex, and there could be up to 70 such complexes in a single vesicle feeding excitation energy to approximately 12 RC-LH1 dimers [49–51]. Given the local curvature imposed by LH2, which on its own can form spherical vesicles [52–54], the mixture of RC-LH1 dimers and LH2 complexes produces ∼50 nm diameter spherical membranes, often called chromatophores, which have been seen in stained electron micrographs of thin sections of *Rba. sphaeroides* cells for decades [55]. The dimeric structure of the core complexes influences the size of chromatophores, and a *Rba. sphaeroides* mutant with LH2, but which makes only core monomers, forms vesicles larger than those found in the WT [56]. The reason why vesicle formation is important for this bacterium is that the curved membrane encloses a small lumen containing perhaps 12 cytochrome *c*_2_ molecules [57], which shuttle back and forth between the RC-LH1 and cyt *bc*_1_ complexes. Cyt *c*_2_ picks up an electron from cyt *bc*_1_ and ferries it to the RC, where the electron reduces the photooxidised RC. The rapid diffusion of cyt *c*_2_ is facilitated by its confinement within the curved membrane vesicle [57]. Despite the functional significance of core dimers for photosynthesis in *Rba. sphaeroides*, many bacteria do not assemble tightly curved vesicles, and some such as *Blc. viridis* contain large planar membrane sheets largely consisting of monomeric RC-LH1 complexes [58]. Other bacteria such as *Rsp. rubrum* have monomeric cores and no LH2 complexes, yet the cell interior is packed with intracytoplasmic vesicles [55]. There is much more to learn about the significance of membrane architectures in various bacteria, and the factors that determine whether they are curved or planar.

## The bacteriochlorophyll and carotenoid pigments of RC-LH1 complexes

Removing the protein scaffolds reveals curved arrays of pigments, which absorb solar energy and feed it to the central RC to initiate charge separation. The geometry and regularity of these light-harvesting arrays are directed by the antenna polypeptides, and details of the pigment binding sites will be presented in the next section on the structures of αβ pigment–protein modules. This, and other topics related to RC-LH1 structure and function, are also covered in a recent review [59]. Here, we can see that the wealth of core complex structures reveals several classes of pigment organisation. Figure 2 shows examples of each, namely the closed 32-BChl *a* ring (*Rps. palustris* RC_3_-LH_16_), open 30-, 28- and 28-BChl *a* rings (*Rba*. *veldkampii* RC_3_-LH_15_-X, *Rps. palustris* RC_3_-LH_14_-W, and *Rba. sphaeroides* RC_3_-LH_14_-XY, respectively), the closed 34-BChl *b* ring (*Blc. viridis*), the 104-BChl *a* double ring (*Gem. phototrophica*) and the 56-BChl *a* core complex dimer (*Rba. sphaeroides*).

The two key features of these arrangements are the close packing of light-gathering pigments in the curved arrays, and the large separation between each array and the central RC cofactors. The extensive contacts between the transmembrane domains of LH1 αβ polypeptides forms a curved scaffold that binds a series of BChl pigments. This curvature is inherent to the LH polypeptides, so circular, elliptical and spiral LH1-only complexes can be imaged by EM [60] and AFM [61,62]. Most of the LH structures bind BChl *a*, but *Blc. viridis* uses BChl *b*; in each case the C17 carbon is esterified with phytol, but unusually the BChl *a* in *Rsp. rubrum* LH1 is esterified with geranylgeraniol [63,64]. Regardless of these variations, the BChls are arranged in a similar fashion and are spaced so closely, with Mg-Mg distances less than 10 Å, that there are overlaps between C/E and A rings of adjacent BChl macrocycles, within and between αβ subunits, respectively. The arrangements of pigments within subunits are covered in more detail in the next section and in Figure 3. The LH1 rings can be thought of as a series of αβBChl_2_Crt subunits, but so well packed that BChls in neighbouring subunits are brought together rather more closely than those within a αβ subunit. The overall effect is to create a continuous array of overlapping BChls (Figure 2). The Q_y_ transition dipoles of the two BChls within a subunit are arranged in opposing directions in the plane of the membrane, and the Q_x_ transitions for the whole ring are aligned perpendicular to the membrane plane; see Figure 2I for a diagram of the Q_x_ and Q_y_ transition dipoles. This arrangement, together with the macrocycle overlaps, induces the excitonic coupling responsible for most of the absorption characteristics of LH1 BChl *a* pigments, which are red-shifted to at least 870 nm relative to the ∼770 nm maximum for the monomeric pigment in solvent. Figure 2 shows that the BChls in each LH1 complex conform to this basic arrangement, underlining the importance of excitonically coupled pigment arrays for light harvesting and energy transmission. Thus, an excited state at any position in the LH1 ring is shared with neighbouring pigments in tens of femtoseconds [65–67]. Figure 2 also shows that there are variations on this theme; for example, BChl-BChl distances are somewhat shorter for complexes that exhibit a larger red shift relative to the absorption of the monomeric pigment. The RC-LH1 complex of *Blc. viridis* (Figure 2D) provides an extreme example, and the environment and organisation of BChl *b* molecules in this LH1 ring impose an unusually large red shift in absorption of ∼220 nm, in relation to the ∼100 nm shift for complexes from *Rsp. rubrum* and *Rba. sphaeroides*, for example. Figure 3 displays the absorption spectra of all complexes discussed in this review. The pigments in *Blc. viridis* are BChl *b*, which has an ethylidene group at the C8 position, rather than the ethyl group found in BChl *a*, so monomeric BChl *b* in solvent absorbs ∼25 nm further to the red than BChl *a* [68]. The αβ_17_ LH1 ring brings together 34 BChl *b* pigments, with the shortest intra-subunit (8.8 Å) and inter-subunit (8.5 Å) Mg-Mg distances reported for a bacterial light-harvesting complex [4]; Figure 2H compares the Mg-Mg distances for BChls from *Rba. sphaeroides* and *Blc. viridis* complexes. The chemical structure and proximity of the BChl *b* pigments, as well as the stability afforded by the outer ring of γ-polypeptides, contribute to the 1,018 nm absorption maximum observed for the complex within the native photosynthetic membrane; there is a blue shift to 1,008 nm when the RC-LH1 complex is solubilised in detergent, as in Figure 3. Thus *Blc. viridis* is able to occupy a unique spectral niche above 1,000 nm, beyond the 920–980 nm absorption of water, and it gains access to a source of energy denied to bacteria that utilise BChl *a* [39].

As with the survey of RC-LH1 architectures in Section 3, the double-ring (RC-dLH) complex from *Gem. phototrophica* merits special mention, although not particularly for its inner LH1 ring, which is similar to other αβ_16_ LH1 complexes that bind 32 BChls and 16 carotenoids. However, LH1 is encircled by the unique LHh complex (Figure 2E), which binds an outer array of BChl and carotenoid pigments [32]. Details of the binding sites are presented in the next section but here we note that the LHh ring has two functionally important features. Each αβ subunit in this 24-fold outer ring binds a dimer of BChls, one monomeric BChl and one carotenoid, so LHh substantially increases the light-harvesting capacity of the *Gem. phototrophica* complex by contributing an extra 72 BChls and 24 carotenoids. Also, the hydrogen bonds to dimeric BChls found in the inner LH1 ring are absent from LHh BChl dimers, which raises site energies and blueshifts the absorption of the paired BChls to 816 nm. The higher energy allows these B816 BChls to act as energy donors to the inner LH1 ring. Furthermore, the addition of 24 binding sites for monomeric BChls, reminiscent of the B800 BChls of LH2 complexes [70,71], creates a B800/B816/B868 energy cascade that favours transfer of excitations from the outer LHh ring to the RC, via LH1, aided by the close, ∼2.5 nm separation between outer and inner ring BChls [32].

Carotenoids are the other major pigments found in all RC-LH1 complexes and their contribution in the 400–550 nm wavelength range compensates for the lack of BChl absorption in this spectral region (Figure 3). Carotenoids are also important structural elements in bacterial LH complexes [72,73], and are required for efficient dimerization of the *Rba. sphaeroides* RC-LH1 complex, for example [74], although LH1 tolerates the absence of these pigments [75–77]. Finally, carotenoids perform an essential photoprotective role by quenching harmful excited triplet states of (B)Chls, which otherwise could generate reactive and damaging oxygen species such as singlet oxygen [78]. There is some conflict between the light-harvesting and photoprotective roles of carotenoids, which centres on the number of alternating single and double bonds, *N*, also called the conjugation length. Smaller values of *N* produce blue-shifted carotenoids capable of donating absorbed light energy to BChls, and when *N* is between 7 and 9 the efficiency of energy transfer is above 90% [79,80]. Carotenoids with larger *N* values are more suited to a dissipative function, because their triplet energies are low enough to quench BChl *a* triplets, although this comes at the cost of energy transfer efficiency [81]. It appears that nature has opted for a compromise that allows light harvesting but with an emphasis on dissipation, and all carotenoids in Figures 2 and 3 are *N* ≥ 9.

In nearly all RC-LH1 complexes each pair of BChls is associated with one carotenoid with only one exception, the LH1 complex of *Rba. sphaeroides*, where two carotenoids are bound to each αβBChl_2_ subunit [33] (see the next section on the structures of αβ pigment–protein modules), which enhances light-harvesting capacity and may enhance photoprotection. The rationale proposed for these extra LH1 carotenoids, and it relates to the exchange of quinones and quinols across the barrier that LH1 creates between the enclosed RC and the external membrane environment [82] as covered in the later section on quinol export. The light-harvesting and photoprotective modes of carotenoids both require close proximity to BChls [79], and for the RC-LH1 monomer of *Rba. sphaeroides*, for example, each spheroidene is within 3–4 Å of a BChl [33]. These hydrophobic pigments lie across the membrane bilayer, slanted so they make contacts with a particular subunit, and with the next one round the ring. Thus, carotenoids confer physical stability on the complex, and numerous interactions with the macrocycles and phytol tails of the BChls foster efficient energy transfer. In the case of *Rba. sphaeroides*, displacement of the phytol of the α-bound BChl creates enough room for each αβBChl_2_ subunit to bind an extra carotenoid with an inverted orientation, which is in van der Waals contact with the other carotenoid. This intimate series of connections between pigments brings together the π-conjugated carbon-carbon double bond systems of the carotenoids within and between subunits and with intervening, excitonically coupled BChls [33].

The short, ∼4–10 Å distances between carotenoids and BChls, and between LH1 BChls, contrast sharply with the large separation between LH and RC pigments of 35 Å or more seen in all structures in Figure 2. The inverted sixth power dependence of the energy transfer rate on the distance between donor and acceptor [83] leads to energy transfer times of 40–50 ps from the LH1 BChls to the the two excitonically coupled RC BChls, often called the ‘special pair’ and given the label P [84,85]. This jump from the LH1 complex to the RC is therefore a major contributor to the overall trapping time of ∼60–70 ps, which is the duration for exciton migration through the antenna network, transfer to the RC and the initial charge separation [86,87]. Nevertheless, such trapping times are still far faster than the nanosecond radiative decay of the BChl excited state, so the overall trapping efficiency can approach 95%, and is not unduly affected by the LH1-RC separation. At the same time, there are positive benefits to holding LH1 and RC pigments apart and maintaining a distance of tens of Å between them, as pointed out by Noy *et al*. [88]. They show that a minimum distance of 24 Å is required to ensure that BChls in the encircling LH complex are held ‘at arm’s length’ from central RC cofactors to avoid damage by cation radicals in the RC electron transfer chain, which could persist for 25 μs. A typical closest approach between an LH1 BChl and P is ∼32 Å in the case of *Rps. palustris* RC_3_-LH1_14_-W (Figure 2C) [27]; this distance ranges between 39 and 49 Å for the closed αβ_16_ ring from *Rps. palustris* RC_3_-LH1_16_ in Figure 2A. Such separations are more than enough to shield LH pigments from oxidation by the strongly electropositive (BChl)_2_^+^ special pair in the RC, while still permitting energy transfer to the RC on a timescale compatible with efficient energy trapping. The same considerations also apply to other closed αβ_16-17_ rings, including those not shown in Figure 2.

Of the closed-ring complexes, *Rsp. rubrum* offers an intriguing variation; the structure at 2.5 Å resolution [25] shows that there is an extra BChl *a*-GG (BChl *a* with an unreduced geranylgeranyl tail) molecule that bridges the gap between LH and RC pigments, positioned 26.6 Å from the pair of BChl *a*-GG pigments attached to the second LH1 αβ subunit, and 22.2 Å from the RC special pair (Figure 2G). This BChl *a*-GG pigment therefore has the potential to act as an intermediary between complexes, accelerating energy transfer to the RC. However, it sits alone, forming a ligand with a nearby phosphatidylglycerol molecule and making a series of hydrogen bonds directly with the RC H-subunit and with the second LH1α subunit, and indirectly via a network of water molecules. Comparison with monomeric BChl *a* in LH2 complexes [89,90] suggests that this lone BChl *a*-GG would have an absorption maximum near ∼800 nm, which is energetically ‘uphill’ from the 875 nm-absorbing BChls in LH1. Onward energy transfer to the RC would be favourable, so this intriguing pigment could act as a conduit for energy transfer to the RC; however, this suggestion is not supported by transient absorption experiments on RC-LH1 complexes from *Rsp. rubrum* (with the extra pigment) and *Rba. sphaeroides* (with no extra pigment), and in both cases the time constants for energy transfer were 30–40 ps [84]. The possibility that the additional BChl *a*-GG participates in photoprotection cannot be ruled out, but experimental evidence for this role is currently not available.

**Figure 9:**
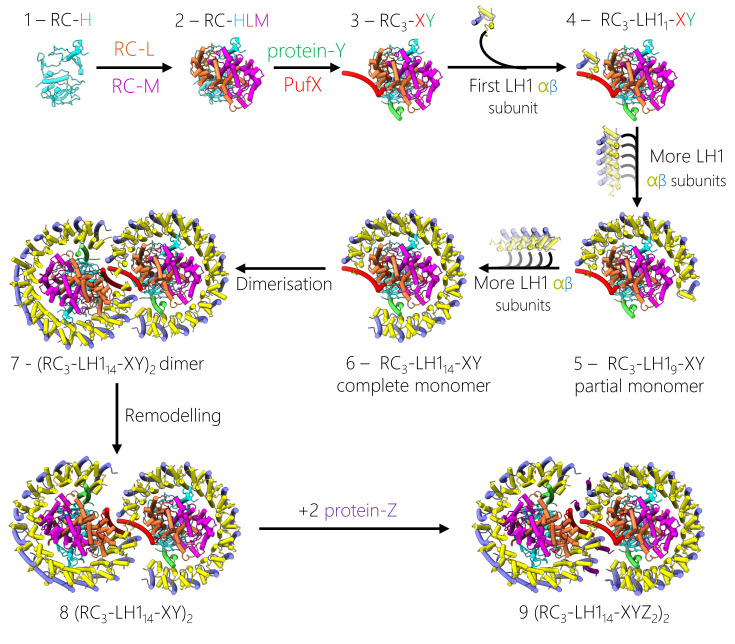
Possible assembly sequence for the dimeric RC-LH1 complex of *Rba. sphaeroides* Assembly stages 1–9 are based on the events proposed in [37,165]. They denote a series of assembly intermediates, viewed from their periplasmic sides and starting with (1) the RC-H subunit, then (2) the binding of RC-L and RC-M subunits. In (3) PufX and protein-Y are recruited, acting as a start signal for assembly of the (RC_3_-LH1_14_-XYZ_2_)_2_ complex and providing an anchoring point for building the LH1 ring, starting with the arrival of the first LH1 αβBChl_2_Crt_2_ subunit (4). (5) More LH1 subunits are added, with the RC acting as a template for correct sizing of the LH1 ring; this assembly intermediate has tightly bound LH1 subunits at positions 7–9, with the shortest EET distance from LH1 BChls to the RC special pair of BChls. (6) Encirclement of the RC is complete, leaving a gap for quinone traffic to and from the RC, and creating the RC_3_-LH1_14_-XY monomer complex. (7) The first stage of dimerization, promoted by C-terminal contacts between each PufX and its opposing LH1 β14 partner. (8) The next stage forms a dimer with two rings of BChls seamlessly connected for excitation sharing between the two halves of the structure. (9) Two copies of protein-Z are recruited. LH1β is in blue, LH1α in yellow, PufX in red, RC-H in cyan, RC-M in magenta and RC-L in brown. BChl and carotenoid pigments have been omitted for clarity.

Figure 2C shows examples of open RC-LH1 complexes with αβ_14_BChl_28_ and αβ_15_BChl_30_ rings, from *Rba. sphaeroides* and *Rba. veldkampii*, respectively. Given that 16 or 17 LH1 αβ subunits are required to enclose a RC [4,37], these αβ_14-15_ rings create an opening that improves the flow of quinone/quinol traffic to and from the RC Q_B_ site. The presence of a single gap likely reflects the requirement for an uninterrupted assembly sequence for the LH1 ring (see Figure 9). Also, one gap in the LH1 ring apparently allows sufficient LH1 subunits to feed energy to the RC while creating a portal for enabling quinols and quinones to pass between the RC and the nearby cyt *bc*_1_ complex [91]. All open LH1 rings have a single gap, which is located at the same location relative to the central RC; this structural feature appears to be an example of convergent evolution. The role of this interruption in the ring, and the way that polypeptides such as PufX, protein-Y and protein-W maintain this gap, will be discussed in the section on exporting quinols. Here, we only consider the possible effects of opening the LH1 ring on LH1-RC energy transfer, which could arise because the free ends of the LH1 arc acquire some freedom either to bend away from or move towards the central RC, changing the LH1-RC energy transfer distance somewhat. The αβ_15_BChl_30_ complex from *Rba. veldkampii* represents a minimal interruption to the closed ring and the open ends of the arc of LH1 subunits nearest the RC Q_B_ site curl towards the RC slightly, decreasing the distance to the RC primary donor, relative to the closed ring for the RC_3_-LH1_16_ complex of *Rps. palustris*, for example, from 38.9 to 38.2 Å (Figure 2C). Similarly, a comparison of open (αβ_14_BChl_28_) and closed (αβ_16_BChl_32_) rings for the RC_3_-LH1_14_-W and RC_3_-LH1_16_ complexes of *Rps. palustris*, respectively, showed that LH1 subunits 12-14 of RC_3_-LH1_14_-W move slightly towards the RC, shortening the distance to the RC primary donor from 38.9 to 32.1 Å. However, there was little consequence for excitation energy transfer (EET) from LH1 to the RC, with time constants of 40 ± 4 and 44 ± 3 ps for the RC_3_-LH1_14_-W and RC_3_-LH1_16_ complexes, respectively [27]. Opening the LH1 ring of the monomeric RC_3_-LH1_14_-X complex of *Rba. sphaeroides* has the opposite effect, and LH1 subunits 11–14 are pushed away from the RC by the two transmembrane helices (TMHs) of protein-Y, which is sandwiched between the inner face of LH1 subunits 11–14 and the RC, adjacent to the Q_B_ site (Figure 2C). As a result, there is quite a large variation in the Mg-Mg distances from LH1 BChls to the RC special pair of BChls; BChls bound to LH1 subunits 7–9 that lie distal to the Q_B_ site are 38.3 Å from the RC, but this distance lengthens to 50.7 Å for BChls bound to subunit 14 [33]. Such an increase could slow down energy transfer to the RC 5-fold, which would have an adverse effect on the overall trapping time by lengthening it hundreds of picoseconds. However, in practice there is likely to be no penalty for widening the arc of LH1 subunits; the overlapping macrocycles and aligned Q_Y_ dipoles of LH1 BChls are configured for ultrafast energy transfer [65–67], so it would take far less than a picosecond for an exciton to migrate from subunit 14 to subunits 7–9. From there, there is a shorter path for EET to the RC, thereby circumventing any possible difficulties with BChls bound to subunit 14. Thus, the *Rba. sphaeroides* LH1 complex offers a degree of specialisation, in which the preferential delivery of energy to the RC from subunits 7–9 gives subunits 11–14 the freedom to move away from the RC, stabilised by protein-Y, in order to optimise quinone traffic.

The dimeric *Rba. sphaeroides* LH1 complex builds on the advantages of the monomer by creating an extended path for excitations to migrate between the two halves of the complex. As Figure 2F shows, there is no gap in the S-shaped array of 56 BChls because of the precise way that the two sets of 28 BChls are brought together. As already pointed out for the monomer there is some limited overlap between the C/E rings of the BChl macrocycles within an LH1 subunit, and the A rings of BChls on adjacent subunits also overlap, but remarkably this continuity is maintained across the join between the rings. Thus, the A rings of BChls bound to the LH1 α1 and α1’ polypeptides (belonging to the other half of the dimer) are still overlaid at the intersection, and the 9.3 Å Mg-Mg distance for the α1 and α1’ BChls is only 0.5 Å longer than the normal Mg-Mg spacing within each monomer [36]. The only pigments that reflect the steric problems in bringing together two monomers are the interface carotenoids, which must accommodate the presence of two PufX polypeptides. As a result, LH1 subunits 1 and 1’ each bind only one carotenoid, rather than the normal two. This is no hindrance to the energy transfer across the interface though, because the BChl overlaps across the join ensure continuity of excitonic coupling. Hence, a dimer offers an advantage over a monomeric complex by creating the possibility for excitation sharing between the halves of the dimer [92]. At higher light intensities, where there is a greater chance that one of the dimer RC traps is closed by photochemical activity, an exciton can move rapidly to the other half of the dimer to gain access to the other RC [87].

## The structures of **αβ** pigment–protein modules that form (αβ)_n_ LH1 complexes

Decades ago, Zuber and colleagues chemically sequenced LH1 polypeptides isolated from a wide variety of purple phototrophs. Then, it became clear that the α and β polypeptides are very similar, and could be aligned to an invariant histidine residue suggested to form the ligand to a bound BChl [93]. This residue fell within a span of 20–25 residues, correctly predicted to form an α-helix that traverses the photosynthetic membrane and that is flanked by N- and C-terminal domains lying on the surface of the cytoplasmic and periplasmic sides of the photosynthetic membrane, respectively [93]. Later, models of LH1 polypeptides incorporated the results of site-directed mutagenesis experiments that showed hydrogen bonds from C-terminal Trp or Tyr residues to BChl C3 acetyl carbonyls [94,95], and also verified the histidine ligand to the BChl Mg ion [96]. The C-terminal hydrogen bonds necessitated a bend in the LH1 α-polypeptide as it emerged from the membrane bilayer [97]. The first high-resolution structure of a RC-LH1 complex [5] showed that the structural and BChl binding features of α- and β-polypeptides had been correctly predicted, and now we have a wealth of RC-LH1 structures that are all built using the same basic αβBChl_2_Crt subunit, but with some interesting and important variations.

So similar are these subunits that only a few are presented in Figure 4, which illustrate variations on the αβBChl_2_Crt theme typified by the *Rps. palustris* RC_3_-LH1_16_ complex: *Tch. tepidum* (Ca^2+^ binding site in each αβ_2_BChl_2_Crt subunit); *Blc. viridis* (an outer γ-polypeptide is attached to all but one of the 17 αβBChl_2_Crt subunits); *Rsp. rubrum* (has BChl-GG rather than BChl-phytol); *Rba. sphaeroides* (extra carotenoid to form a αβBChl_2_Crt_2_ unit); *Rfx. castenholzii* (the LH1 ring comprises αβBChl_3_Crt units, with the extra BChl adding an 800-nm absorption band), and *Gem. phototrophica* (the outer LHh ring comprises αβ_2_BChl_3_Crt units that add 800- and 816-nm absorption bands). In some cases, the membrane-extrinsic regions of LH polypeptides are not fully traced in the density maps. Several factors can contribute, such as the likely flexibility of these regions or proteolytic cleavage. The *Rps. palustris* αβ heterodimer in the upper panels of Figure 4 shows the features predicted earlier, in which an αβ pair of transmembrane polypeptides coordinates a dimer of BChls through histidine residues and a carotenoid, in this case spirilloxanthin, lies across the membrane making close contact with both polypeptides and with the α-bound BChls. There are also hydrogen bonds between α-Trp43 and β-Trp47 and the C3-acetyl carbonyl of each BChl, which are partly responsible for the red shift of BChls to 875 nm relative to their ∼770 nm absorption in solvent. Removal of the α-Trp43 hydrogen bond by mutagenesis blue-shifts the *Rba. sphaeroides* complex to 853 nm and, separately, removing the β-Trp48 hydrogen bond produces a ∼5-nm blue shift [94,95,98]. A doubly mutated complex was too unstable to assemble but these mutagenesis studies show that, relative to monomeric BChl, approximately 80 nm of red shift arises from the binding of each BChl to its cognate polypeptide and, crucially, excitonic coupling of BChls within and between αβBChl_2_Crt subunits.

The separation of the LH1 complexes into αβBChl_2_Crt subunits in Figure 4 is somewhat arbitrary, given that BChls in neighbouring subunits are brought together more closely than those within an αβ subunit, as noted in Section 4. Accordingly, three successive subunits, in this case 7–9, are shown in Figure 4B to emphasise the continuity between BChl dimers made possible by the association of neighbouring subunits. Figure 4C,D show views perpendicular to the membrane from the cytoplasmic and periplasmic sides of the complex; in each case there are hydrogen bonds within and between the soluble loops of adjacent subunits that help to stabilise the formation of curved oligomeric arrays. BChl-BChl, Crt-protein and Crt-BChl interactions within the membrane also make major contributions to the stability of the LH1 complex. Analysis of other structures reveals similar stabilising features and although they will not be specified here, the general principles are that a series of intricate pigment–protein, pigment–pigment and protein–protein bonding arrangements form LH1 subunits and hold them together to create curved (αβ)_n_ LH1 complexes.

Several variants of the αβBChl_2_Crt subunit are shown in the lower panels of Figure 4, which build on this basic arrangement through the addition of more pigments or another polypeptide. One well documented modification is the ring of 16 bound Ca^2+^ ions found in the *Tch. tepidum* complex, in which side-chains from α and β polypeptides, as well as two water molecules, create a six-coordinate binding site near the periplasmic face of the membrane [29]. This particular feature appears to confer structural stability [99], which is beneficial for growth of this thermophile at 55°C, but it also contributes to the 915-nm absorption, which is red-shifted relative to the more usually observed 875 nm [100]. *Trv*. strain 970 (not shown in Figure 3) also binds a circular array of Ca^2+^ ions, but in this case a hydrogen bonding network involving the BChl *a* C3-acetyl group, α-Trp47, α-His48 and Ca^2+^ locks the complex together [28]. A further contribution is made by the extended C-termini of LH1 α-2 and α-4, which interact with the RC C- and M-subunits on the periplasmic face of the complex (see the later section on lipids and RC–LH1 interactions). Taken together, these interactions, which are not found in other complexes, push the LH1 absorption maximum to 960 nm, the most red-shifted BChl *a* absorption in an RC-LH1 complex. *Alc. tepidum* also binds Ca^2+^, but only in six of the 16 subunits. This is because this organism produces its LH1 from multiple copies of its αβ polypeptides, some with Ca^2+^ binding sites and others lacking this feature [30].

However, the most red-shifted absorption, to 1,018 nm, is found in the *Blc. viridis* RC_4_-LH1_17_-γ_16_ complex, for which a αβγBChl_2_Crt subunit is shown in Figure 4F. Several features of this subunit were proposed to account for the red-shifted absorption maximum, including the presence of BChl *b* rather than BChl *a*, and having a larger than normal ring of 17 subunits and therefore 34 BChls. The closer packing of BChls, with shorter Mg-Mg distances and increased overlap between pyrrole rings (Figure 2H) contributes to the red shift, as does the recruitment of the γ-polypeptide [4]. As the panel in Figure 4F shows, each γ-polypeptide packs closely between β-polypeptides, and collectively 16 such interactions round the LH1 ring, comprising 32 hydrogen bonds to α- and β-polypeptides, enforce a level of stability and rigidity that constrains the LH1 ring and contributes to the red shift of the BChl *b*-Q_y_ absorption band. The subunit of *Rsp. rubrum* represents a minor variation, in which the BChl macrocyle is esterified with geranylgeraniol rather than the more usual phytol (Figure 4G) [25]. Comparison with the *Rps. palustris* BChl-phytols shows that the presence of three extra double bonds does not appear to constrain the positions adopted by the tail.

The three variations in the bottom row of Figure 4 involve the addition of an extra pigment to a subunit. Most conservatively in this row, just one more carotenoid, Crt2, has been added to form an αβBChl_2_Crt_2_ subunit in *Rba. sphaeroides* (Figure 4H) [33] (here, we use the nomenclature Crt1/2 rather than the CarA/B used in [33,36]). Crt1 corresponds to those found in subunits from other complexes and it interlinks between subunits, running from the N-terminal region of the β-polypeptide on the cytoplasmic side of the complex and then lying across the α-bound BChl on the adjacent subunit. Displacement of the α-BChl phytol tail makes room for binding the second carotenoid, in an inverted orientation relative to Crt1. The position of Crt2 is also shifted towards the periplasmic side of the membrane, such that C3 of Crt2 sits by C20 of Crt1. The consequence of accommodating this extra carotenoid is the creation of a series of new carotenoid-carotenoid, carotenoid-BChl and BChl-BChl contacts that bring together the π-conjugated C=C bond systems of the Crts within and between subunits. The α-bound BChl is positioned between Crt1 and Crt2 so, given the excitonic coupling between BChls in the LH1 ring, both the BChl and Crt populations are seamlessly connected for strong energetic coupling and rapid energy transfer.

The final two examples, from the *Rfx. castenholzii* LH1 and the *Gem. phototrophica* outer LHh ring, both involve the addition of an extra BChl *a* pigment that produces an αβBChl_3_Crt subunit (Figure 4I,J). In each case this additional BChl lies towards the cytoplasmic face of the complex, some distance from the excitonically coupled ring of BChls, so in both instances it is essentially monomeric with an absorption maximum near 800 nm. In this respect this BChl resembles the B800 pigments found in LH2 complexes, although it does not adopt the same orientation in the *Rfx. castenholzii* LH1 complex. In LH2 complexes from *Rps. acidophila* and *Rba. sphaeroides*, for example [71,73], the B800 lies approximately in the plane of the membrane whereas the B800 in the *Rfx. castenholzii* LH1 complex is tilted approximately 60° from this plane. β-His26, β-Trp14 and keto-γ-carotene form the binding site; in *Rba. sphaeroides* LH2 the B800 binding site also involves a nearby carotenoid molecule, but the central ligand to B800 is a carboxyl oxygen of the modified N-terminal α-carboxymethionine [71]. A B800-880 nm energy transfer time of 2 ps was measured for the *Rfx. castenholzii* LH1 complex [101] so the addition of an extra BChl, absorbing where there is little contribution from the main LH1 ring, would be expected to confer some benefits in terms of light harvesting. Even more advantageous is the addition of an outer LH ring in the case of *Gem. phototrophica*; this extra complex adds 24 B800 pigments as well as an excitonically coupled ring comprising 48 B816 BChls. Thus, this outer LHh complex makes two more absorption bands available for light harvesting, both sitting at a higher energy than LH1 BChls and thereby creating an energy cascade [32]. LHh performs the same functions as LH2 complexes do in phototrophic bacteria such as *Rba. sphaeroides* and *Rps. palustris*, but it lacks any flexibility to respond to light intensity by increasing or lowering the number of LH2 units. Nevertheless, *Gem. phototrophica* has found an economical way of acquiring significantly more absorption for each RC by using the same β-polypeptide twice, for both the inner and outer LH rings, in combination with a PufA2 α-polypeptide that is only distantly related to other LH1 α-polypeptides [32]. Figure 4J shows an αβBChl_3_Crt subunit from LHh, with an all-*trans* carotenoid, gemmatoxanthin. As with the monomeric BChls in the *Rfx. castenholzii* LH1 complex the B800 pigment is tilted well away from the plane of the membrane, in this case by nearly 90°, but more importantly the 18.5 Å distance to the nearest outer ring B816 BChl allows a 0.4 ps time constant for B800-B816 energy transfer [32]. The outer LHh assembly is stabilised by a series of inter-subunit interactions through hydrogen bonds that link each α-polypeptide to its neighbour at their C termini, as well as hydrophobic interactions with gemmatoxanthin; a lipid, 1-palmitoleyl-2-myristoyl-*sn*-phosphatidylethanolamine, also helps to lock adjacent subunits together.

## The focus of harvested solar energy – the reaction centre

The function of antenna-RC complexes in photosynthesis is to gather photons, and direct their energy to the RC where it is used to drive a charge separation event that ultimately reduces an acceptor. This reduced acceptor transiently stores the energy in a chemical form, which is subsequently used further downstream to generate a proton-motive force and ATP, or to reduce substrates. All the RCs enclosed by the various antenna rings shown in Figure 1 are designated type II, with quinones as the final electron acceptors, as also seen for photosystem II of oxygenic phototrophs [102]. In type I RCs iron-sulphur centres are the electron acceptors. Recent progress in cryo-EM studies of RC-LH1 complexes has revealed a new set of RC structures; some, such as those from *Rsp. rubrum, Rps. palustris, Trv*. strain 970, *Tch. tepidum* and *Alc. tepidum*, and *Rba. veldkampii* are similar to known RC complexes, and others, from *Rba. sphaeroides* and *Blc. viridis*, provide cryo-EM versions of structures already determined by X-ray crystallography [103,104]. However, recent cryo-EM studies of RC-LH1 complexes reveal three RC structures, from *Rfx. castenholzii, Gem. phototrophica* and *Rba. veldkampii*, that exhibit some novel features. There is a large body of literature on RCs, so the purpose of this section is to present a short, comparative summary of RCs in some of the recent structures of RC-LH1 complexes. For detailed accounts of mechanistic aspects, quantum coherence and photoelectrochemical applications of RCs the reader is referred to recent reviews and articles, and references therein [105–109].

The three-subunit RC complex from *Rba. sphaeroides* (Figure 5A) provides the basic blueprint for the type II RCs found in purple phototrophs. The TMHs of the L and M subunits create a hydrophobic cage that houses the electron transfer cofactors; their identities, positions, distances and orientations do not differ significantly between complexes (Figure 5) nor are they altered fundamentally, even in the more distantly related PSII complexes [102]. All the examples in Figure 5 show a branched network of cofactors, with BChl and bacteriopheophytin (BPhe) pigments, two quinones, and an Fe^2+^ ion present. Apart from the *Rfx. castenholzii* complex, all characterised RCs bind a carotenoid in the M-subunit to protect against triplet excited states of P. There are two closely apposed BChls, coloured in green in Figure 5, that superficially resemble the pairs of LH1 BChls bound to LH1 αβ subunits (see Figure 4). However, in the RC it is the A rings of the BChl macrocycles that overlap, and they are brought together more closely, with a Mg-Mg distance of 7.8 Å. The particular forms of P found in various RCs are further labelled with their ground state absorption maximum, which in the case of the BChl *a*-containing *Rba. sphaeroides* RC leads to P870, and to P960 for the BChl *b*-containing RC from *Blc. viridis*, for example. Despite the large distance from the nearest LH1 BChls, which is 38.3 Å from those bound to LH1 subunits 7–9, the Q_y_ dipoles of LH1 and P870 BChls sit at approximately the same level in the plane of the membrane (Figure 2A) and there is nearly complete spectral overlap between donor and acceptor pigments. So, despite the distance constraints, there is a forward rate constant of 35–50 ps for LH1-to-RC energy transfer, which is compatible with efficient trapping if P870 is in its ground state and able to receive energy from the surrounding antenna. However, it is accepted that, as well as the forward process of energy transfer from LH1 to the RC, there can also be detrapping of an excited state from the RC back to the surrounding LH1 complex. Indeed, repeated detrapping and retrapping events have been envisaged [83]. Retrapping could occur either at the same RC or, following transfer to an adjacent LH1 complex, at another RC; excitation sharing within a dimeric RC-LH1 complex [92,110] increases the chances of such retrapping events. Whether excited states move from LH1 to the RC, or in the reverse direction, the same relatively large distance is involved and these processes have time constants of tens of ps, slower than formation of P* then the charge separated state P^+^H_A_^−^ in a few ps. Thus, the likelihood of detrapping is affected by the population of states P, P*, P^+^H_A_^−^ and P^+^Q_A_^−^, which affects the equilibrium between an excited state in LH1 and the P* and P^+^H_A_^−^ states in the RC [111]. The effects of open and closed RCs on trapping/detrapping are discussed in [112]; for *Rsp. rubrum*, the authors find an ‘escape probability’, of ∼14% with open RCs and ∼66% with closed RCs.

Despite the apparent symmetry of the branched cofactor network, only those on one side of the complex, designated branch A, are used for electron transfers (Figure 5A). The kinetics of formation of these states have been studied in many laboratories and are outside the scope of this summary; instead, the reader is referred to reviews of this topic, such as [105,109,113]. There is also a rich literature on the history of photosynthesis, and the emergence of the concept of RCs from the work of Duysens, Clayton and others, summarised in [114,115]. Once an exciton reaches P from the nearest LH1 BChls the excited state P* is formed in femtoseconds, which initiates a cascade of successively slower electron transfers that culminate in the millisecond formation of a reduced quinone acceptor (Figure 5A). Within 3 ps of forming P* an electron is transferred to the neighbouring monomeric BChl, designated as B_A_, creating the P^+^B_A_^−^ radical pair. The replacement of the central Mg by two protons in the next acceptor, BPhe (designated H_A_), lowers the midpoint redox potential of this pigment so it can act as an acceptor for the electron from B_A_^−^, forming the P^+^H_A_^−^ radical pair. The state P^+^Q_A_^−^ is formed within 200 ps, again favoured by a drop in redox potential as well as by the 10.1 Å distance between H_A_ and Q_A_. ET to form P^+^Q_B_^−^ is a great deal slower and takes ∼100 μs [109], and now other processes are initiated. One of these is the replacement of the electron ‘lost’ from P by reduced cyt *c*_2_, which docks onto the periplasmic face of the RC and resets the system from P^+^ to P in a few microseconds so that the RC can undergo a second charge separation [116–120]. The ability of Q_B_^−^ to accept a second electron is therefore important, and two successive proton-coupled electron transfers form QH_2_ [121]; which can leave the Q_B_ site and exit the RC-LH1 complex so it can reduce the cyt *bc*_1_ complex [51]. The export/import of QH_2_/Q from the confines of the RC Q_B_ site and passage across the LH1 ring are covered in the section on quinol export.

The processes of LH1-RC EET, then forward electron transfer within the RC, then resetting the system for a second charge separation, and finally export of QH_2_ are interlinked [88]. The interplay of energy and redox levels, as well as inter-cofactor distances, allow the RC-LH1 complex to cope with varying incident light intensities while minimising detrapping from the RC to LH1 and the oxidation of LH1 pigments by P^+^, and at the same time avoiding recombination of P^+^B_A_^−^, P^+^H_A_^−^ and P^+^Q_A_^−^ radical pair states to the ground state [88]. Rapid and efficient turnover of the RC therefore requires the timely docking/undocking of cyt *c*_2_ and the Q_B_-site quinone, which ferry electrons to and from the RC, respectively. The formation of dissociable QH_2_ is conserved in all type II RCs, but there are different arrangements on the electron donor side. Figure 5B shows that some RCs, exemplified here by *Blc. viridis* and *Gem. phototrophica*, have arrived at a different solution for a reducing the P^+^ state, which involves a four-heme C-subunit ‘hardwired’ to the periplasmic face of the RC. In *Blc. viridis* the midpoint redox potentials of these hemes do not smoothly follow a sequence of high to low, and instead they form a ‘rollercoaster’ [109] that nevertheless supplies electrons to P^+^ on a single microsecond timescale (reviewed in [122]). Following electron transfer to the RC, the tetraheme cyt is reduced by accepting an electron from a mobile carrier such as cyt *c*_2_, a high potential iron-sulphur protein (HIPIP) or auracyanin. Recently a structure of HIPIP bound close to heme-1 was determined for the *Tch. tepidum* RC-LH1 complex [123].

The striking feature of the *Blc. viridis* RC is the incorporation of BChl *b* and BPhe *b*, which redshifts RC absorption bands and produces a P960 special pair (Figure 5B) [124]. Relative to the 1,018 nm absorption of the surrounding BChl *b*-containing LH1 complex, P960 lies energetically ‘uphill’ from the surrounding antenna although at room temperature this is no barrier to the transfer of excitation energy [86,125]. Another feature of the *Blc. viridis* RC is the use of different quinones at Q_A_ and Q_B_, which are menaquinone and ubiquinone, respectively [109]. The *Rfx. castenholzii* RC has a BPhe on the inactive B-branch in place of the monomeric BChl found in most other RCs, and both Q_A_ and Q_B_ are menaquinone (Figure 5C). Like the *Blc. viridis* RC it has a four-heme cyt attached in this case with an N-terminal extension that traverses the membrane, allowing it to associate with LH1 subunits but also to interrupt the ring, which has 15 LH1 subunits [35]. This cyt extension appears to function like PufX in *Rba. sphaeroide*s in that it prevents closure of the LH1 ring, creating a gap that could allow the passage of quinones between the RC and the exterior of the complex. Close by are two other TMHs; one of these, designated TM7, is an independent single helix that is proposed to be a hitherto unidentified component or a product of proteolysis of RC-L or -M subunits. There is no RC-H subunit and, unusually, RC-L has six TMHs rather than the normal five; the extra helix, TM1, occupies the position normally adopted by the single RC-H TMH in other complexes. Finally, there is another TMH, TM-X, that sits in the gap close to the 15^th^ LH1 subunit [35]. An N-terminal extension has also been found in the four-heme cyt subunit of the *Rpi. globiformis* RC (not shown in Figure 5), which traverses the membrane and interacts with RC and LH1α polypeptides. This C-subunit therefore plays roles in electron transfer and stabilisation of the complex. Although the transmembrane extension does not interrupt the LH1 ring, it is suggested to be a progenitor of the PufX found in *Rhodobacter* species [31].

The *Gem. phototrophica* RC (Figure 5D) resembles the template seen for *Rba. sphaeroides* (Figure 5A) and *Blc. viridis* (Figure 5B), in terms of the arrangement of cofactors, but there are some highly unusual structural features. Within the four-subunit RC, the RC-H subunit is divided into two parts comprising Hc, a membrane-extrinsic domain (∼19.9 kDa) on the cytoplasmic side of the complex, and Ht, a ∼7.7 kDa polypeptide corresponding to the transmembrane section of RC-H in *Rba. sphaeroides* [32]. More remarkable is the presence of surface-lying polypeptides on each face of the complex that appear to stabilise interactions between the RC and the double-ring antenna (see also Figure 8C). Together, two RC-S polypeptides extend from two opposing sides of the LH1 ring towards the central RC; they start at the N-terminal region of LH1 at either subunit 8 or subunit 16, and then lie in the plane of the membrane across the periplasmic surface of the RC so they almost meet in the middle of the complex. RC-S binds to the surface regions of RC-M, RC-C, and RC-L subunits through a series of hydrogen bonds, so they help to hold the inner LH1 ring to the central RC. On the other side of the complex, there is a RC-U polypeptide comprising a pair of helices that forms a hairpin structure; RC-U clamps the LH1 ring to the RC by attaching at one end through hydrogen bonds to the N-terminal regions of LH1 subunit 13 and at the other to the RC-Hc subunit [32]. There are no counterparts to RC-S and RC-U in any other structure determined to date and they appear to have evolved to impart an extra level of stability and resilience to the double-ring RC-LH1_16_-LHh_24_ complex. The final example of a RC variation is found in *Rba. veldkampii* (Figure 5E), which in most respects is very similar to RCs in *Rba. sphaeroides* and *Rps. palustris*, for example. However, there is an extra BPhe, situated on the inactive B branch of the cofactor network, 7.0 Å from Q_B_ and 7.7 Å from the B branch BPhe, edge-to-edge for the conjugated part of each molecule. The function of this third BPhe is unknown at present [34].

## Exporting the quinols produced by the RC-LH1 complex

All RC-LH1 complexes exist to capture light, and to transiently store the energy as a quinol. The high efficiencies of energy transfer and trapping are of little value unless the final product, a quinol at the Q_B_ site, is able to escape from the confines of the surrounding LH1 ring and diffuse on millisecond timescales to the cyt *bc*_1_ complex. This section will cover the variety of strategies adopted by RC-LH1 complexes for enabling quinone diffusion.

Two rounds of RC photochemistry produce a Q_B_ quinol, but this is only one of several quinones to consider and at any one time there are quinols from previous RC turnovers traversing the surrounding LH1 antenna, en route to the cyt *bc*_1_ complex. There are also several quinones arriving from the cyt *bc*_1_ complex, which ensure a steady supply of substrate for impending reductions at the Q_B_ site; this mixed population of quinols and quinones forms an intramembrane pool, part of which is sequestered within the RC-LH1 complex. Thus, as many as 15 quinones per RC can be extracted from purified RC-LH1 complexes [126]. At room temperature, these molecules can occupy multiple locations as they jostle within the confined space between the RC and the inner wall of the surrounding LH1 complex, obscuring structural analysis of their positions. However, sample preparation for cryo-EM or X-ray crystallography appears to trap at least some quinones in certain positions consistently enough for them to appear in density maps. Remarkably, structurally resolved quinones from several RC-LH1 complexes can be superimposed, even though they were purified from many species of proteobacteria, using a variety of methods. Apart from the expected Q_A_ and Q_B_ sites, the overlaid quinones in Figure 6A reveal four more sites for quinone occupancy labelled Q1-Q4 and all mapped onto a single RC, in this case from *Rps. palustris*. The consistent locations of these sites within diverse RC-LH1 complexes likely reflect snapshots of a generic diffusion path for quinols as they undock from the RC Q_B_ site, move within the space that separates the outer face of the RC and the inner face of the LH1 complex, and finally traverse the LH1 barrier. Q1-Q4 could map the positions of local minima in an energy landscape, in which quinones are trapped by rapid freezing; disorder in the density map prevents further assignments for the majority of the quinones quantified by extraction [126], but developments in cryo-trapping protocols and image processing methods could reveal more quinones in future.

Figure 6B shows an enlarged view of site Q2 with quinones from each structure in a unique colour. In the *Rps. palustris* structures, interactions of the quinone head group with L-Trp143 and L-Gln88 were observed, and multiple sequence alignments show these two residues are strongly conserved in bacterial type II RCs. The coincidence of their positions could indicate a location where quinones transiently reside prior to passage across the LH1 ring. The first such exit point was originally assigned for the *Blc. viridis* complex as Q_P_ [4], and here this quinone forms part of the cluster now termed Q3 (Figure 6A). Figure 6C shows the position of Q2 relative to the opening in the RC_3_-LH1_14_ -W complex of *Rps. palustris*, and Q3 is just visible in Figure 6D behind a BChl phytol chain due to a small gap in the LH1 ring, one of many in the closed RC_3_-LH1_16_ complex. For reference, Figure 6E shows the corresponding position in the RC_3_-LH1_14_-XY complex from *Rba. sphaeroides*; here, the LH1 ring comprises an array of αβBChl_2_Crt_2_ LH1 subunits, each with an extra Crt that obscures the small gap for quinone export allowed by αβBChl_2_Crt units (see Figure 6D). Figure 6F shows a series of projection views of representative RC-LH1 complexes that summarise possible routes for quinone traffic. The thicknesses of the dashed lines provide rough approximations of the likelihood of quinone diffusion, based on gaps in the LH1 antenna identified in the structures, and the LH+ and Q+ designations provide rough indications of the capacity of each complex for transferring excitation energy and quinones, illustrating the conflicting requirements for these processes. At one extreme, the double, closed rings of in the RC-LH1 complexes from *Gem. phototrophica* present the most challenging barrier for export of quinols from the RC. However, the structure shows that α-α polypeptide spacings for the outer LHh ring are 16.4 Å, larger than the 14.8 Å for the LH1 ring, so the inner LH1 structure could be the major obstacle for quinols and quinones, as for other closed rings found in *Rsp. rubrum, Rps. palustris, Trv*. strain 970, *Tch. tepidum* and *Alc. tepidum* (Figure 1). In these structures it is assumed that ‘breathing’ motions and small gaps between LH1 polypeptides could allow quinone traffic [127]. A ‘free’ quinone, designated Q_F_ in the *Gem. phototrophica* complex [32] and corresponding to Q3 in Figure 6A, lies close to the inner wall of LH1 adjacent to weak B800 density in the outer LHh ring. Another structurally resolved quinone sits near another possible exit point in the inner ring that coincides with even weaker B800 density. The lower B800 densities in the outer LHh complex could stem from quinone diffusion at these points in the ring, giving rise to local disorder, or from a Q-channel forming where there is low occupancy of this BChl binding site. Figure 6F (top left) shows a possible diffusion path for quinones in *Gem. phototrophica*; the LH and Q designations reflect the extra light-harvesting capacity conferred by the outer LHh ring, as well as the restricted possibilities for quinone diffusion.

In comparison with the *Gem. phototrophica* complex, other complexes with single rings (Figure 6F) sacrifice some light-gathering ability while improving the prospects for quinone diffusion. However, in *Blc viridis* γ-subunits block 16 of the 17 potential quinone pores, imposing a single exit point at the only position with no γ-polypeptide [4]. In the *Rpi. globiformis* LH1 5/16 binding sites lack a γ-like outer polypeptide [31], and this lower occupancy is expected to permit more movement of quinones across the LH1 barrier. The wild-type strain of *Rps. palustris* is unusual in assembling open RC_3_-LH1_14_-W and closed RC_3_-LH1_16_ complexes, both of which are apparently able to sustain photosynthetic growth [128], providing a useful structural comparison. Figure 6F depicts quinone traffic via the ‘breathing’ motions in the RC_3_-LH1_16_ complex and such small gaps between polypeptides are likely found in other closed ring systems. The RC_3_-LH1_14_-W complex represents a degree of specialisation, with one part of the closed ring, and its light-harvesting capacity, sacrificed to create an open channel for unobstructed quinone diffusion. Protein-W appears to prevent binding of a 15^th^ and 16^th^ LH1 subunits, counteracting the natural tendency of the assembly system to fully enclose the RC (see the later section on assembly of the RC-LH1 complex). The open RC_3_-LH1_14_-XY complex of *Rba. sphaeroides*, shown in Figure 6F in its monomeric form, embodies yet more specialisation [33]. The structure of the fully encircled PufX-minus complex (not shown) has been determined, revealing a complete ring of 17 LH1 αβ subunits, so the gap in the native structure can accommodate another three LH1 subunits [37]. This gap is formed by the PufX polypeptide, as also seen in the *Rba. veldkampii* complex. However, *Rba. sphaeroides* has recruited another component, protein-Y (also called protein-U in [38]), which forms an internal quinone channel between its inner face and RC-L, providing more access to the Q_B_ site. On the other side of protein-Y the two TMHs lie against the inside face of the LH1 α polypeptides at positions 13 and 14, acting as a spacer that holds the flexible, free end of the LH1 arc away from the RC [33]. AFM of membranes and complexes from an LH1-only mutant showed that, in the absence of any restraints, LH1 subunits are linked so flexibly that they can form curved arrays of varying sizes and shapes [61,62]. Together, protein-Y and PufX prevent LH1 subunits 11–14 from curving towards the RC, which would constrict the ring opening and hinder quinone diffusion. Another component, protein-Z, was identified in the structure of the dimeric RC-LH1 complex from *Rba. sphaeroides*, (RC_3_-LH1_14_ -XYZ_2_)_2_, but its structural and functional roles are uncertain [36].

In summary, the RC-LH1 structures show how the core complexes from *Rps. palustris, Gem. phototrophica*, *Blc. viridis*, *Rpi. globiformis* and *Rba. sphaeroides*, used here as examples of open, closed, single and double rings balance the opposing requirements for harvesting light and exporting quinols. An extreme solution to this problem would be to dispense with the LH1 ring, and indeed a RC-only mutant of *Rba. sphaeroides* requires no quinol/quinone channel, since it is open to the membrane bilayer; however, this mutant harvests little light, which retards photosynthetic growth [129,130]. This situation improves for mutants of LH1 unable to form a complete ring [131]. These structures illustrate the trade-off between packing the LH1 antenna with too many light-absorbing pigments, which could impede quinone diffusion, and assembling too few LH1 subunits round the RC, which would hinder growth under low light conditions.

## Lipids and RC–LH1 interactions

### Protein-lipid interactions

Dezi et al [126] showed that the RC-LH1 complex of *Rba. sphaeroides* contains over 150 lipids, approximately half of which were cardiolipin, with 24% phosphatidylglycerol, 12% phosphatidylethanoamine, and 14% phosphatidylcholine. Despite their likely disordered conformations, several lipids have been resolved within RC-LH1 structures, along with detergent molecules. Many of these lipids and detergents are found sequestered between the LH1 ring and the RC, in the gaps between the transmembrane regions of the proteins. The *Rps. palustris* RC_3_-LH1_16_ structure [27] has been selected here as an example of a closed ring complex; Figure 7A shows that several cardiolipins are located on the cytoplasmic side of the membrane in the region of the RC-H TMH [27]. In Figure 6B, lipids from three more selected complexes, from *Tch. tepidum* (RC_4_-LH1_16_), *Rba. veldkampii* (RC_3_-LH1_15_-X) and *Rba. sphaeroides* (RC_3_-LH1_14_-XY) [29,33,34], are overlaid on those in *Rps. palustris*, emphasising their similar positions. Cardiolipin is the predominant lipid identified in these structures, but we note that assignment of cardiolipin is relatively straightforward as it is the only lipid with four hydrophobic tails, allowing it to be identified easily from its distinctive shape. Cardiolipins have been previously observed in crystal structures of RCs [132], and as already mentioned biochemical studies have shown that RC-LH1 complexes are enriched in cardiolipin [126]. These structural and biochemical findings suggest that cardiolipin binding sites are strongly conserved. On the lumenal face of the complex several more lipids, assigned as phosphatidylcholine and phosphatidylglycerol in the *Rps. palustris* structure (Figure 7C), are commonly observed (Figure 7D). However, the identity of these lipids is more variable across the range of RC-LH1 structures, possibly as a result of the lipid type being less well conserved, the difficulty in reliably assigning structurally similar phospholipids with two tails from their density, or the variable replacement of lipids with detergent molecules. Lipids near the RC-H TMH are in excellent agreement on both leaflets of the membrane, but on the distal side of the RC their positions are more variable, reflecting the variation in structure across the family of RC-LH1 complexes in this region, and because greater flexibility of the lipids here may aid quinone diffusion through the lipophilic environment they provide. Analysis of the two *Rps. palustris* structures identified many well-conserved RC and LH residues with hydrogen bonds to phospholipids. These specific interactions and ‘gap-filling’ properties of the lipids within the LH1 ring suggest they play a major role in stabilising interactions between LH1 and the RC. In addition to the lipids inside the LH1 ring, lipids and detergents are often seen intercalating with the LH1 subunits on the outside of LH1, particularly on the lumenal side of the complex (Figure 7C,D). These lipids may provide additional stabilising interactions and provide an interface between the RC-LH1 complex and the membrane bilayer in which they are embedded.

Lipids have been found to play additional important structural roles in RC-LH1 complexes. The transmembrane signal peptides of most RC-C subunits are cleaved during export to the periplasm, and in some complexes, such as the RC-LH1 from *Tch. tepidum*, the cysteine residue at the export cleavage site is modified with a covalently bound lipid [29]. This group provides a hydrophobic anchor that helps to tether the RC-C subunit to the rest of the complex (Figure 7E). This modification is common to membrane associated cyts that lack a TMH. The dimeric complex of *Rba. sphaeroides* shows another potentially important role played by a lipid [36]; in this case the local resolution was unusually high allowing assignment during the modelling process as sulfoquinovosyl diacylglycerol (SQDG). On each side of the dimer the lipid head group is hydrogen bonded to PufX arginine residues 49 and 53 (not shown), both of which are essential for dimerization, whilst the SQDG lipid tails extend into each half of the monomer making extensive lipid–protein and lipid–pigment interactions at the dimer interface (Figure 7F). There could be another lipid in this region, which is labelled as Lipid-2 in Figure 7F. This has been suggested to be an ornithine lipid, which is known to be present in *Rba. sphaeroides* [133,134].

### Protein–protein interactions between the RC and LH1

In all RC-LH1 complexes a series of protein–protein interactions binds the LH1 ring to the RC. Using the *Tch. tepidum* RC_4_-LH_16_ complex [29] as an example, the outer face of the transmembrane A helix of RC-L makes predominantly van der Waals contacts with the inner transmembrane face of α2 of the second LH1 subunit (Figure 8A, lower left panel). There are also interactions between the soluble loops of LH1 subunits 1–3 and surface-exposed residues of RC-L. In closed ring structures these can extend to the LH1 α that is absent from open rings (α16 in *Tch. tepidum*). The TMH interactions can be found in all structures, but the surface interactions are somewhat variable because there are differences in the structures of the LH1 extrinsic regions. TMHs A and B of the M-subunit make extensive van der Waals interactions with α8-9 of LH1, with additional surface interactions between M and LH1 α subunits 7–12, predominantly on the lumenal face of the membrane, and a single interaction with β8 on the cytoplasmic face (Figure 8A, upper right panel). Again, the transmembrane interactions are observed in most structures, whereas the membrane surface interactions are variable. Interactions with the RC-H TMH are limited to a few polar interactions and hydrogen bonds on the cytoplasmic and lumenal surfaces with α5 and α6 of LH1 (Figure 8A, top left panel). There are also interactions between the soluble region of H and the cytoplasmic loops of LH1 α2-5 (Figure 8A, lower left panel). Similar interactions involving the RC-H subunit are present in all RC-LH1 complexes except *Rfx. castenholzii*, which lacks an H-subunit. In complexes with a C-subunit there are typically minimal interactions with three hydrogen bonds between C and LH1α subunits 12, 14 and 15 (Figure 8A, lower right panel). The limited interactions seen in some RC-LH1 complexes containing C-subunits may explain why some RCs have evolved without a C-subunit with no impact on the integrity of the rest of the complex. However, in C-subunits with TMH anchors there are additional interactions with LH1, although these are variable in the *Rfx. castenholzii* and *Rpi. globiformis* complexes because their RC-C TMHs bind in different locations [31,35]. In the *Rpi. globiformis* complex the N-terminal domain of the C-subunit forms a series of bonds on the cytoplasmic face of the complex with RC-M and RC-H residues, and via a polar bond to an LH1 α-polypeptide.

Some LH1 complexes have additional unique interactions between the RC and LH1. In several species the α polypeptides have extended C-terminal regions that associate with the C-subunit (for instance *Trv*. strain 970 in Figure 8B). In this example the extended α subunits (α2 and α9) are encoded by one of the additional *puf* operons that produces LH1 subunits, resulting in a heterogeneous LH1 complex [28]. *Alc. tepidum* also contains extended α subunits but those that interact with RC-C are in different locations (α13 and α15) around the ring [30]; the corresponding interactions are with α10-12 and α15 in *Rpi. globiformis* [31]. As already noted in Section 6, supernumerary subunits in the *Gem. phototrophica* complex interact with both RC and LH1 [32]. One of these subunits, designated RC-S, extends across the lumenal face of the complex interacting with LH1 on opposite sides of the ring at subunits 8 and 16, forming hydrogen bonds with RC-M, RC-C, and RC-L subunits. On the cytoplasmic side of the complex, the RC-U polypeptide forms hydrogen bonds to the N-terminal regions of LH1 subunit 13 and at the other end to the RC-Hc subunit. Thus, the extrinsic RC-S and RC-U polypeptides bind the LH1 ring to the RC (Figure 8C). Figure 8D illustrates another type of interaction that helps to bind LH1 to the RC. In *Rhodobacter* species the PufX protein holds the LH1 ring open, and it interacts with the RC-L subunit on the lumenal face of the RC; also, the PufX TMH interacts with the TMHs of the first LH1 αβ pair towards the cytoplasmic side of the membrane (Figure 8D) [33]. PufX is present in RC-LH1 complexes of *Rba. sphaeroides* and *Rba. veldkampii* and it is presumed that PufX proteins of other *Rhodobacter* species, such as *Rba. capsulatus*, are similar. In addition to PufX, *Rba. sphaeroides* has an additional, unique subunit called protein-Y (or protein-U) [33]. Protein-Y binds the RC-L subunit on the cytoplasmic and lumenal sides of the complex leaving a gap between itself and the transmembrane region of the RC. The outer face of protein-Y interacts with α13 and α14 of LH1, providing a set of stabilising RC-LH1 interactions that lock LH1 into a conformation that assists quinone diffusion to and from the RC Q_B_ site (Figure 8D), as discussed above.

## Assembly of RC-LH1 complexes

### General aspects of membrane and photosystem assembly

Facultative photoheterotrophs can dispense with the need for light, and can turn to respiration as a source of energy. This interesting property was noticed many years ago in bacteria such as *Rba. sphaeroides* (then *Rps. spheroides*), *Rsp. rubrum* and *Rba. capsulatus* (then *Rps. capsulata or capsulatus*) [135–138]. Early investigations into photosystem assembly took advantage of this property of *Rba. sphaeroides*, by using high levels of aeration to suppress pigment biosynthesis and formation of intracytoplasmic membranes in dark-grown cultures. RC-LH1 assembly and development of intracytoplasmic membranes could be initiated in the dark by lowering the level of oxygen [139–143]. Further control can be exerted by modulating light levels in photosynthetically grown cultures of *Rba. sphaeroides* [24,144,145]. These approaches showed that formation of RC-LH1 complexes dominates the early stages of development, followed by assembly of LH2 complexes round the RC-LH1 complexes to form mixed membrane regions, then further accretion to form LH2-only domains [143,146–148], the size of which is inversely proportional to the light intensity in photosynthetically grown cultures [24,145]. It was possible to fractionate cell extracts and isolate an ‘upper pigmented band’ (UPB), containing membrane sites where photosystem assembly and membrane invagination are initiated [149]. This role was verified by pulse-chase experiments, which also showed that they were ‘hotspots’ for the biosynthesis of proteins [149,150] and BChls [145], also supported by a subsequent mass spectrometry study [151]. Spectroscopic investigations showed that these immature UPB membranes were not fully established in terms of energy and electron transfers [152–154], and AFM showed that the curved, contact lens-shaped morphology seen in EM of negatively stained samples is imparted by the presence of a few RC-LH1 dimers [150]. The prior bending of the membrane by the RC-LH1 dimer favours the subsequent incorporation of LH2 complexes [56], which also have membrane-curving properties [54]. Monte Carlo simulations show how the shapes and curvature-inducing properties of RC-LH1 and LH2 complexes encourage partitioning into separate domains, the presence of which was verified by AFM, native gel electrophoresis and linear dichroism [45,155]. The end result of the stepwise assembly sequence is a budded intracytoplasmic membrane 50–60 nm in diameter that retains its original site of attachment to the cytoplasmic membrane, or which can detach fully to become an independent photosynthetic ‘organelle’ [150]. Analysis of solubilised complexes on sucrose density gradients [80] and AFM [24,156] show that the (RC_3_-LH1_14_-XYZ_2_)_2_ dimer is the dominant form of this complex in mature chromatophore membranes, and there are approximately 11 in a single chromatophore, along with ∼2 RC_3_-LH1_14_-XY monomers [49]. Thus, these core dimers impart significant curvature to the membrane and tend to associate in groups, surrounded by ∼65 LH2 complexes [49–51].

Early analyses of energy transfer using singlet-singlet annihilation [146,157], and a more recent ultrafast study of live, photosynthetically grown cells of *Rba. sphaeroides* [85], show that an intracytoplasmic membrane vesicle, the chromatophore, can be considered as a single functional unit in terms of energy transfer, trapping and ATP production [49–51]. Extensive computational simulations, based on AFM, mass spectrometry, spectroscopy and EM studies culminated in a 100-million atom-scale model of a chromatophore that accounts for all bioenergetic events, from absorption of light to EET, charge separation at the RC, turnover of the cyt *bc*_1_ complex and production of ATP [51]. Computational models of chromatophores were substantiated by AFM of single, intact chromatophore vesicles [158]. Remarkably, these simulations can be extended to account for doubling times of the bacterium under a variety of light intensities [159].

Although the mature photosynthetic membrane is well characterised, and the UPB sites for initiation of membrane assembly can be isolated, little is known about the provision of carotenoids, BChls and nascent LH and RC polypeptides, and the processes that ensure that they are brought together efficiently. There is an important role in RC-LH1 assembly for LhaA, predicted to be a membrane-intrinsic protein comprising 477 amino acids. The *lhaA* gene lies within the photosynthesis gene cluster (PGC), a ∼50 kb region of the genome where most of the genes related to the biosynthesis, assembly, regulation and function of RC-LH1 complexes are known to reside in a number of phototrophic bacteria (for example, [160–163]). In the PGC of *Rba. sphaeroides lhaA* is situated next to *puhA*, which encodes the RC-H subunit; deletion of *lhaA* abolishes the assembly of most, but not all, LH1 complexes [164]. A combination of spectroscopy, immunoprecipitations, native gel electrophoresis, quantitative mass spectrometry and fluorescence lifetime microscopy shows that LhaA forms oligomers at sites where membrane invagination is initiated, and that it associates with RCs, BChl synthase (BchG), the protein translocase subunit YajC, and the YidC membrane protein insertase [56]. It was proposed that LhaA is part of a membrane nanodomain in which the close proximity of biosynthetic and membrane insertion components promotes coordinated pigment delivery, the co-translational insertion of LH polypeptides, and their folding and assembly to form photosynthetic complexes [56].

### Assembly of the RC-LH1 complex

Despite the uncertainties regarding the formation of RCs, the provision of pigments and the synthesis of αβBChl_2_Crt_2_ subunits, we can propose a sequence of events that culminates in the formation of the dimeric (RC_3_-LH1_14_ -XYZ_2_)_2_ complex of *Rba. sphaeroides*. Indeed, of the many RC-LH1 structures recently determined, the dimeric RC-LH1 from *Rba. sphaeroides* has received the most attention in terms of its assembly pathway, including the sequence of events suggested earlier [165]. The unusual bent structure of the dimer (Figure 1) imposes curvature on the photosynthetic membrane in which it sits, so the assembly of the complex is inseparably linked to the formation of intracytoplasmic membranes. The sequence of events that form the RC and then encircle it with LH1 subunits is not known in detail, but some general points can be made. RC-LH1 complexes are likely built from the inside, RC first, then moving outwards; then, there is a site on the RC where the first LH1 subunit binds, acting as the initiation point for recruiting more subunits [147]. In cases where there is a non-LH1 component, such as PufX in the case of *Rba. sphaeroides* or *Rba. veldkampii*, attachment of this protein creates a specific binding site that acts as the starting point for LH1 assembly. As oligomerisation proceeds and the complex starts to wrap around the RC, the flexibility of the LH1 array [61,62] allows it to conform quite closely to the shape of the RC, fostering specific LH1-RC contacts. In effect, the RC acts as a template for LH1 assembly, and oligomerisation proceeds until either the RC is fully enclosed, or until it comes to a halt because a PufX polypeptide prevents addition of more αβ subunits. The result is either a closed ring or one with a gap, and examples of both are shown in Figure 1.

Figure 9 depicts a possible sequence of events that form the dimeric (RC_3_-LH1_14_-XYZ_2_)_2_ complex from *Rba. sphaeroides*, incorporating the pathway suggested recently [37]. Obtaining structures of intermediates would provide valuable snapshots of this assembly process, and such progress has been made for biogenesis of PSII in cyanobacteria [166], but despite the absence of such structures it is possible to piece together a likely series of steps in the assembly of the *Rba. sphaeroides* dimer complex. The structures of complexes lacking PufX, but still with protein-Y, and of alternative conformations of the dimeric (RC_3_-LH1_14_ -XY)_2_ complex of *Rba. sphaeroides* [37,38], add to the picture of dimer formation. The assembly sequence is assumed to start with the RC-H subunit, based on early immunological analyses [167], but in fact there is little known about how the RC complex forms. A time course of RC-LH1 assembly was studied in a low-aeration cell culture, before the existence of protein-Y was known, which showed that PufX is incorporated into developing core complexes relatively early in the assembly sequence, followed by LH1 [43]. Thus, having arrived at the three-subunit RC at stage 2, it is proposed that PufX and protein-Y polypeptides are the first to attach to their docking sites on the RC; however, protein-Y appears later in the sequence proposed by Cao et al [37]. The C-terminal domain of PufX is proposed to bind initially to the RC-L subunit via an extensive network of hydrogen bonds. The transmembrane region of PufX makes a series of van der Waals contacts with RC-L corresponding to stage 3 in Figure 9. Protein-Y is also proposed to bind to the RC early in the assembly sequence, via H31, W22, F33, L36 on its periplasmic hairpin loop docking with W265, W266, L269 and W271 on the RC-L subunit [33]. We suggest that the first LH1 subunit attaches to the N-terminal domain of PufX, yielding stage 4. Attachment of this first subunit adds only one carotenoid instead of the two in subsequent LH1 subunits; the structure of the dimer complex shows that each of the Crt2 carotenoids is missing from the monomer–monomer interface because of the proximity of PufX polypeptides [36]. Subsequent recruitment of further αβBChl_2_Crt_2_ subunits is likely to progress in a clockwise direction, with reference to Figure 9, stabilised by interactions with the preceding subunit through overlapping BChl macrocycles and carotenoids that interlink neighbouring subunits. No carotenoids were found in the final, 14th subunit.

As detailed in the section on RC-LH1 protein-protein interactions, LH1 subunits at positions 2–9 form a series of bonds with RC subunits; LH1 αβ2-4 are bonded to the RC-L and RC-H subunits, LH1 αβ6 to RC-H, and LH1 αβ8,9 are hydrogen-bonded to RC-M [33]. These RC–LH1 interactions are important, and AFM studies showed that in their absence there is a loss of control of LH1 subunit oligomerisation, in terms of both the number of subunits and their morphology [62]. By the time that nine LH1 subunits have been added a complex has formed with some light-harvesting capacity, and with an open side on the RC that would allow free exchange of quinols and quinones at the Q_B_ site. In theory, assembly could stop at this point with a RC_3_-LH1_9_-XY complex, and indeed mutational and suppressor studies show that RCs with incomplete LH1 arrays can assemble and function [131,168,169]. However, it is reasonable to suppose that there is an advantage in having the extra light-harvesting capacity conferred by adding five more LH1 subunits. As these LH1 subunits are added, and the complex winds round towards the RC Q_B_ site, the LH1 subunits 13 and 14 attach to the outside face of protein-Y. LH1 α-Arg15 residues form hydrogen bonds with Val4, Thr49 and Asn51 of protein-Y and a hydrophobic interface is formed between the transmembrane regions of LH1 α13, α14 and protein-Y [33]. These interactions are consistent with a structural analysis of a complex lacking protein-Y (protein-U in [170]), in which oligomerisation has halted after the addition of 11 LH1 subunits [37]. The intervention of protein-Y widens the arc of LH1 subunits away from the RC, creating a space for quinones and quinols to enter and leave the RC Q_B_ site, and by stage 6 a monomeric RC_3_-LH1_14_-XY complex has formed (Figure 9). Biochemical and AFM analyses show that this monomeric complex is found in membranes, particularly when the level of aeration of the cells produces spheroidenone as the major carotenoid; however, anaerobically grown cells producing spheroidene have mainly dimeric complexes [80]. It has been proposed that the final step in assembly yields an asymmetric complex with differently sized gaps in the LH1 rings [38]. Figure 9 shows two classes of (RC_3_-LH1_14_-XY)_2_ complex structures, both symmetrical, with the stage 8 complex in the sequence proposed to form when the two monomer halves of the class-2 dimer twist [37]. This slight rotation, which was first noticed in dimer-only tubular membranes, is propagated along hundreds of laterally packed dimers creating linear, helical arrays that align along the long axis of the tubes [12]. In the spherical intracytoplasmic membranes found in wild-type *Rba. sphaeroides*, the dominance of LH2 complexes counteracts this tendency of dimers to form long-range helical arrays [45,47]. Recruitment of protein-Z is proposed to occur at stage 9 in Figure 9, forming the (RC_3_-LH1_14_-XYZ_2_)_2_ dimer complex, which is the structure determined in [36]. However, much more work needs to be carried out to define this assembly sequence more precisely.

## Summary and Outlook

By comparing and contrasting the thirteen unique RC-LH1 structures available at the time of writing, this review highlights the remarkable variation in the architectures of these complexes. The variation occurs at the level of the common components, for example, the presence or absence of the RC-C subunit, the structures of the individual polypeptides, and the pigments bound by the RC and LH1 subunits. Further diversity originates from the way in which the individual subunits associate, for example, to produce open or closed LH1 rings, and the presence of additional subunits. Many of these additional subunits appear to have been recruited during evolution and are unique to just a few species of bacteria.

As additional structures of RC-LH1 complexes are determined we expect this diversity to grow, imparting a deeper understanding of how evolution balances the conflicting requirements for maximising pigment density for light harvesting with the need to permit efficient diffusion of quinones between the RC and the quinone pool. Structurally resolved quinones will provide further insights into quinone diffusion across the surrounding LH1 rings, and alternative conformations observed by cryo-EM will provide insight into conformational changes and dynamics occurring in these complexes, which are too often assumed to be static. These structures will also allow the rational design of new RC-LH complexes, either via a mix-and-match approach where components of several RC and LH complexes are combined, or through the *de novo* design of new components inspired by natural variation. The ability to combine near-atomic resolution structures with decades of biochemical and spectroscopic characterisation is adding new depths to our understanding of RC-LH1 complexes of purple bacteria and will lead to many exciting new discoveries in the years to come.

## Note added in proof

Whilst this manuscript was under review two structures of the *Rba. capsulatus* RC-LH1 complex were published. Bracun et al. reported a 3-subunit RC encircled by 15 LH1 subunits, with the ring interrupted by a single copy of PufX [171], whereas Tani et al report a smaller complex with 10 LH1 subunits [172]. The overall architecture of the former structure resembles the RC_3_-LH1_15_-X complex from *Rba. veldkampii*, but each LH1 subunit binds two carotenoids, as found in *Rba. sphaeroides*. In addition, Tani et al observe a lipid-bound BChl within the 10-subunit LH1 ring, in a different location to the additional BChl in the *Rsp. rubrum* structure. Finally, Bracun et al performed variability analysis and showed significant flexibility of the LH1 subunits close to the opening in the LH1 ring, which may be important for quinone exchange via the gap.

## Open Access

Open access for this article was enabled by the participation of University of Sheffield in an all-inclusive *Read & Publish* agreement with Portland Press and the Biochemical Society under a transformative agreement with JISC.

## Abbreviations

AFM: atomic force microscopy
BChl: bacteriochlorophyll
Crt: carotenoid
cyt: cytochrome
EM: electron microscopy
LH: light-harvesting
RC: reaction centre
TMH: transmembrane helix
